# Identification of a hydroxycinnamoyl‐CoA double bond reductase (HDR) affirms multiple pathways for dihydrochalcone formation in apple

**DOI:** 10.1111/pbi.70225

**Published:** 2025-07-09

**Authors:** Susan Schröpfer, Christian Haselmair‐Gosch, Christoph Böttcher, Christian Molitor, Jens Keilwagen, Lukas Eidenberger, Silvija Marinovic, Matthias Hackl, Andreas Spornberger, Benjamin Walliser, Christopher Schlosser, Karl Stich, Annette Rompel, Henryk Flachowsky, Heidi Halbwirth

**Affiliations:** ^1^ Julius Kühn‐Institute (JKI) – Federal Research Centre for Cultivated Plants Institute for Breeding Research on Fruit Crops Dresden Germany; ^2^ Institute of Chemical, Environmental and Bioscience Engineering Technische Universität Wien Vienna Austria; ^3^ Julius Kühn‐Institute (JKI) – Federal Research Centre for Cultivated Plants Institute for Ecological Chemistry, Plant Analysis and Stored Product Protection Berlin Germany; ^4^ Julius Kühn‐Institute (JKI) – Federal Research Centre for Cultivated Plants Institute for Biosafety in Plant Biotechnology Quedlinburg Germany; ^5^ Institute of Viticulture and Pomology BOKU University Vienna Austria; ^6^ Fakultät für Chemie, Institut für Biophysikalische Chemie Universität Wien Vienna Austria

**Keywords:** phloridzin, phloretin, *p*‐dihydrocoumaroyl‐CoA, alternative translation initiation, *Malus* ssp., *Arabidopis thaliana*

## Abstract

Dihydrochalcones are secondary metabolites with manifold dietary and pharmaceutical properties, but with a yet unclear function in plants. The abundance of dihydrochalcones, particularly phloridzin, makes *Malus* ssp. unique in the plant kingdom. The gene(s)/enzyme(s) for the key reaction in phloridzin biosynthesis, the reduction in the C3‐bridge connecting the aromatic rings A and B of chalcones, have long been searched for. To date, two possible pathways to phloridzin have been described, one via the reduction in *p*‐coumaroyl‐CoA and the other via the reduction in naringenin chalcone. In this work, an enzyme from apple leaves, which catalyses the reduction in *p*‐coumaroyl‐CoA, was purified and partially sequenced. The newly identified hydroxycinnamoyl‐CoA double bond reductase (HDR) has not been mentioned in the context of phloridzin biosynthesis so far. Long‐read sequencing and sequence analyses in search of transcriptional and translational variants predicted a *Md*HDR proteoform potentially formed by alternative translational initiation. Heterologous expression of the two *HDR* proteoforms in *E. coli* showed that recombinant HDR is able to reduce *p*‐coumaroyl‐CoA beside other hydroxycinnamic acid CoA esters, whereas other substrates including the corresponding free hydroxycinnamic acids and naringenin chalcone were not accepted. Heterologous expression of the *HDR* proteoforms in *A. thaliana* resulted in the formation of *O*‐hexosylated dihydrocinnamic acids, confirming the functional activity of the HDR as a reductase *in planta*, but no phloretin derivatives were detected. Our findings support the presence of an alternative pathway to the recently described reduction in naringenin chalcone in the biosynthesis of dihydrochalcones.

## Introduction

Dihydrochalcones are secondary metabolites of manifold dietary and pharmaceutical interest. Naturally occurring dihydrochalcones are, for example potent antidiabetic, antifungal, anti‐inflammatory, antimalarial, antibacterial and antineoplastic compounds (Payne *et al*., 2018). In particular phloridzin (phloretin 2′‐*O*‐β‐D‐glucoside), as one of the most prominent dihydrochalcones, is well‐studied for its various physiological activities and pharmacological effects (Shelke *et al*., 2024; Tian *et al*., 2021). In addition, artificial dihydrochalcones are potent sweeteners (Ciçek, 2020; Horowitz and Bruno, 1963).

Dihydrochalcones are closely related to chalcones and flavonoids. They show high structural similarity to chalcones, from which they differ only by a saturated C3‐bridge connecting the aromatic rings A and B (Veitch and Grayer, 2006). Chalcones, the common intermediates in flavonoid biosynthesis, in contrast, have an unsaturated C3‐bridge that is a prerequisite for the formation of the heterocyclic C‐ring of the flavonoid structures (Forkmann *et al*., 1999). Compared with chalcones, which are commonly formed in the plant kingdom, the distribution of dihydrochalcones is quite heterogenous and more than 250 dihydrochalcones from over 46 plant families have been identified (Rivière, 2016). Dihydrochalcones have for a long time been regarded as apple (*Malus* ssp.) specific compounds, since they are abundant in green apple tissue, accounting for up to 80% of the soluble polyphenolic compounds (Hilt *et al*., 2003; Versari *et al*., 1997), mainly represented by phloridzin. Meanwhile, phloridzin was also found in various tissues of over 25 other plant genera (Tian *et al*., 2021). Its physiological role in apple tissue still remains unclear. Involvement in plant defence and plant growth have been suggested (Dare *et al*., 2013a, 2013b, 2017; Gosch *et al*., 2010b; Wang *et al*., 2023; Zhao *et al*., 2024). For a systematic investigation of the relevance of dihydrochalcones in apple, knowledge of enzymes and genes relevant for their formation is essential.

**Figure 1:**
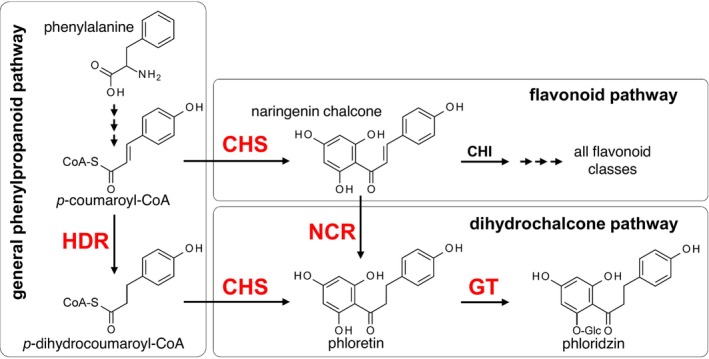
Biosynthesis of phloridzin. The crucial reaction step, the reduction of the double bond, can take place either via the reduction of *p*‐coumaroyl‐CoA to *p*‐dihydrocoumaroyl‐CoA by a hydroxycinnamoyl‐CoA double bond reductase (HDR), or the reduction of naringenin chalcone to phloretin by a naringenin chalcone reductase (NCR). Chalcone synthase (CHS) is involved in both pathways. Glucosylation of phloretin to phloridzin is catalysed by a 2′‐*O*‐glucosyltransferase (GT). Conversion of naringenin chalcone by the chalcone isomerase (CHI) marks the entry point to all further flavonoid classes.

Although phloridzin has long been known to be formed in large amounts in apple tissue, its biosynthesis was investigated more intensively only some 15 years ago (Gosch *et al*., 2009). Two possible pathways leading to phloridzin have been described to date (Figure 1). The biosynthesis can start with the reduction in the *p‐*coumaroyl‐CoA precursor introducing the saturated bond in the C3‐bridge by a hydroxycinnamoyl‐CoA double bond reductase (HDR) leading to *p*‐dihydrocoumaryol‐CoA. This is followed by the formation of the dihydrochalcone phloretin by the common chalcone synthase (CHS) with subsequent glucosylation of the hydroxy group in position 2′, leading to phloridzin, catalysed by a glucosyltransferase (GT). Alternatively, the biosynthesis can start with the reduction in naringenin chalcone by a naringenin chalcone reductase (NCR), which likewise culminates in the final glucosylation step. Several glycosyltransferases from *Malus* ssp. with significant differences in amino acid sequences have been identified to date that can catalyse the glucosylation of phloretin and in some cases also other flavonoid substrates (Dare *et al*., 2017; Gosch *et al*., 2010a, 2012; Jugdé *et al*., 2008; Zhou *et al*., 2017). The step of dihydrochalcone structure formation is catalysed by the common CHS from three molecules malonyl‐CoA and one molecule *p*‐dihydrocoumaroyl‐CoA (Gosch *et al*., 2009). In apple, three *CHS* genes are present, which differ only in a few amino acids. Silencing of the three *CHS* resulted in a reduction in both flavonoid and dihydrochalcone concentrations in apple leaves (Dare *et al*., 2013b). Whereas the involvement of CHS and GT in phloridzin biosynthesis has been characterized in detail, the genes encoding the enzymes for the reduction of the C‐C double bond in the C3‐bridge have long been searched for. Several enzymes that catalyse the reduction in structurally related compounds from other pathways were identified as candidates but could be excluded after recombinant enzyme testing (Adamu *et al*., 2019; Gosch *et al*., 2009). The best candidate until then was a human medium chain acyl‐CoA dehydrogenase (NCBI database sequence 1EGE_A), but no ortholog was found in the apple of the Velasco *et al*. (2010) genome. Two enoyl reductases (ENRL‐3 and ENRL‐5) from apple (Dare *et al*., 2013a) and a hydroxycinnamoyl‐CoA double bond reductase (*Md*DBR) (Ibdah *et al*., 2014) have been suggested to enable *p*‐dihydrocoumaroyl‐CoA formation *in planta*. So far, neither of these reductases has been convincingly shown themselves to play a key role in dihydrochalcone biosynthesis. Studies on ENRL‐3 and ENRL‐5 provided ambiguous results. Transient overexpression of *ENRL‐3* but not of *ENRL‐5* led to an increase in phloridzin concentrations in tobacco while functional activity of the recombinant enzyme could only be demonstrated for ENRL‐5. However, RNAi apples with reduced expression of *ENRL‐3* and *ENRL‐5* showed decreased phloridzin concentration to 66% of wild‐type level. The *Md*DBR was biochemically characterized in detail, including a high‐resolution crystal structure (Caliandro *et al*., 2021). The recombinant protein, however, accepted *p‐*coumaraldehyde and *p*‐hydroxybenzalacetone as substrates, but neither *p*‐coumaric acid nor *p‐*coumaroyl‐CoA, and is therefore most likely out of contention. In the past, most research focussed on the double bond reduction in the level of *p*‐coumaroyl‐CoA. However, an alternative pathway via the reduction in naringenin chalcone was very recently proposed (Yauk *et al*., 2024). It was demonstrated that NCR(s) mediate dihydrochalcone biosynthesis in apple leaves, which opened up the possibility of multiple pathways being involved. In this study, we present the identification of a HDR candidate enzyme by purification from apple leaves and partial sequencing. This resulted in the identification of a *trans*‐2‐enoyl‐CoA reductase that converts *p‐*coumaroyl‐CoA with high specificity into *p‐*dihydrocoumaroyl‐CoA. Moreover, ectopic expression of the HDR encoding gene from apple in transgenic *Arabidopsis* plants resulted in an increase of glycosylated *p*‐dihydrocoumaric acid, dihydrocaffeic acid and dihydroferulic acid.

## Results

### Purification of candidate enzyme(s) possessing HDR activity

The youngest fully developed leaves from apple trees were collected and used for protein isolation and purification, which was optimized upon results from HDR activity tests. During the optimization of the extraction process, a huge impact of the usage of detergent was observed. While little HDR activity could be detected without any detergent, the application of 1%–4% (v/v) Triton X‐114 drastically increased the detected HDR activity of the crude extract. This, however, was strongly dependent on the raw material/extraction buffer ratio. Using a higher amount of raw material and a lower volume of extraction buffer, the expected aqueous two‐phase separation (Molitor *et al*., 2015) did not develop, accompanied by the loss of any HDR activity. Increasing the detergent percentage of the extraction buffer to 10% (v/v), however, resulted in a reproducible extraction method with high HDR enzyme activity. Frequently, a wax‐like layer, presumably composed of cuticle and Triton X‐114, was observed at high detergent concentrations after centrifugation. The optimized purification protocol consisted of four cationic exchange chromatography runs, one based on SP Sepharose Fast Flow, and the others on Mono S 5/50 GL (Figure S1a–d). Finally, the active fractions were pooled and subjected to SDS‐PAGE analysis (Figure S1e), and the most prominent band was subjected to protein identification by HPLC–ESI–MS/MS.

### Protein identification and isolation of the corresponding gene

**Figure 2:**
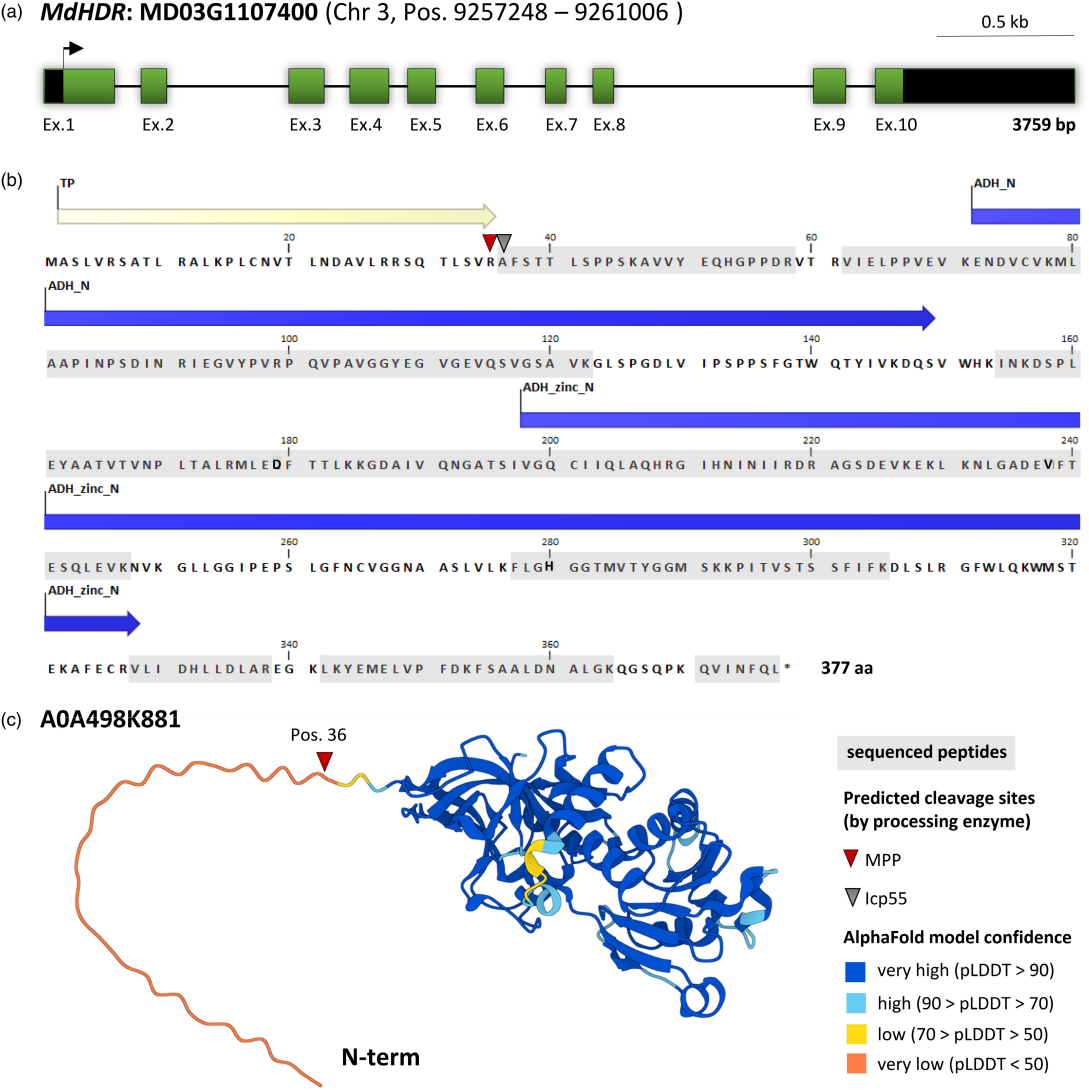
Identified hydroxycinnamoyl‐CoA double bond reductase (HDR) from *Malus* × *domestica*. (a) The identified candidate gene *MdHDR* (MD03G1107400) is located on chromosome 3 in the apple reference genome GDDH13 V1.1. The scheme represents the annotated gene model including exon sequences (named Ex with ascending order) as box, intron sequences as line, coding sequences in green, untranslated sequences in black and the position of the start codon by an arrow. (b) Protein sequence of the canonical proteoform *Md*HDR. Peptides found with LC‐ESI‐MS/MS after tryptic digestion (Table S1) are highlighted in grey. Conserved protein domains were identified using Pfam domain search and are marked in blue within the protein sequence: Zinc‐binding dehydrogenase (ADH_zinc_N, Pos. 198–328, PF00107.30); Alcohol dehydrogenase GroES‐like domain (ADH_N, Pos. 72–149, PF08240.16). A mitochondrial presequence was predicted using MitoFates with a probability value of 0.860, and the cleavage positions of processing enzymes MPP (mitochondrial processing protease, Pos. 36) and Icp55 (mitochondrial intermediate cleaving peptidase 55 kDa, Pos. 37) are indicated by triangles. The protein sequence N‐terminal of the cleavage site is refered to as the mitochondrial transit peptide (TP). (c) The 3D structure of *Md*HDR generated by AlphaFold (https://alphafold.ebi.ac.uk/entry/A0A498K881) is depicted and model confidences are given in the legend.

Identification of the purified enzyme was accomplished via HPLC–ESI–MS/MS. The peptides identified by MS sequencing after tryptic digestion are shown in Table S1. To identify the corresponding gene, tBLASTn analysis was performed on the Genome Database for Rosaceae website (www.rosaceae.org) using the apple reference genome GDDH13 V1.1 (Daccord *et al*., 2017). The BLAST hits mapped to a single gene (MD03G1107400) on chromosome 3 (Figure 2a), hereinafter referred to as *MdHDR*. The obtained peptide sequences cover approximately 65% of the encoded protein and are highlighted in grey (Figure 2b). The corresponding protein sequence in the NCBI database, XP_008358101.1 (E‐value 0.000e+0; 99.20% identity), is described as a ‘probable *trans*‐2‐enoyl‐CoA reductase, mitochondrial isoform X2 [*Malus × domestica*]’. The *Md*HDR is composed of 377 amino acids and a Pfam domain search revealed the presence of a zinc‐binding dehydrogenase and an alcohol dehydrogenase GroES‐like domain (Figure 2b). Compared with the canonical *Md*HDR proteoform (XP_008358101.1), no peptide sequences from MS sequencing mapped to the N‐terminus, suggesting this region is not part of the active enzyme. Sequence analysis with MultiLoc28 (Höglund *et al*., 2006) and MitoFates (Fukasawa *et al*., 2015) predicted an N‐terminal mitochondrial transit peptide (TP) presequence and identified a proteolytic cleavage site in front of the alanine at position 36 (Figure 2c). This aligns with the coverage of the sequenced peptides.

### Gene expression of *MdHDR* in different apple tissues and cultivars

**Figure 3:**
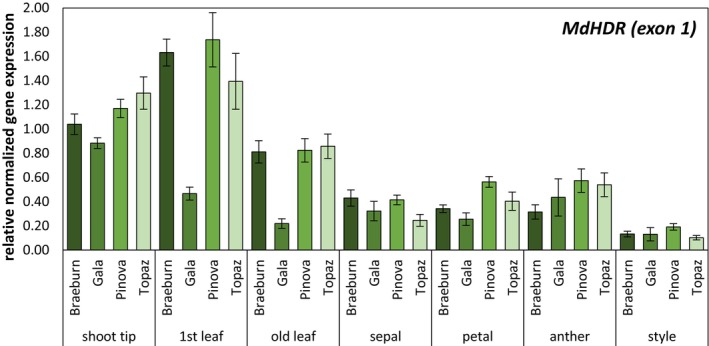
*MdHDR* gene expression in different apple cultivars and tissues. qPCR was performed with pooled samples of different tissues collected from apple trees of ‘Braeburn’, ‘Gala’, ‘Pinova’ and ‘Topaz’ using a primer pair amplifying sequences of exon 1 of *MdHDR*. The mean value of three technical replicates is represented as bar and the standard deviation in given as error bar.

qPCR analyses were conducted to quantify the normalized, relative *MdHDR* gene expression in shoot tips, young and old leaves as well as in floral organs (e.g. petals, sepals, anthers and styles) from four apple cultivars (Figure 3). For normalization of gene expression, the expression levels of the established reference genes (Reim *et al*., 2019; Schröpfer *et al*., 2018), *MdEF1α*, *MdEF1β* and *MdACT7*, were used, which had been selected from a pool of six using the software tool RefFinder (Xie *et al*., 2023). Considering all tissue types and cultivars for reference gene selection, the geometric mean ranking values were 1.73 for *MdEF1α*, 1.86 for *MdEF1β*, and 1.86 for *MdACT7*. The quantification of *MdHDR* mRNA level was carried out using two primer pairs (Table S2). The first pair, MdHDR(ex1)‐FW/REV, amplifies a PCR product from exon 1, with the forward primer binding to the 5′ untranslated region (UTR) sequence and the reverse primer targeting a region encoding for the TP (Figures 2a and 3). *MdHDR* expression was detected in all tested tissues, with higher expression observed in leaf tissue and shoot tips compared to all floral organs. Overall, the highest level of *MdHDR* expression was found in young leaf tissue. The cultivars ‘Braeburn’, ‘Pinova’, and ‘Topaz’ displayed similar expression levels, while ‘Gala’ exhibited lower *MdHDR* expression in both young and old leaves. The relative expression levels observed were validated using the second primer pair (Figure S2). This pair (MdHDR(ex1/2)‐FW/REV) consists of an intron‐spanning forward primer that binds to the border region of exon 1 and exon 2, along with a reverse primer located in exon 2. The specificity of both *MdHDR*‐specific qPCRs was confirmed by Sanger sequencing.

### Catalytic activity of recombinantly expressed HDR

**Figure 4:**
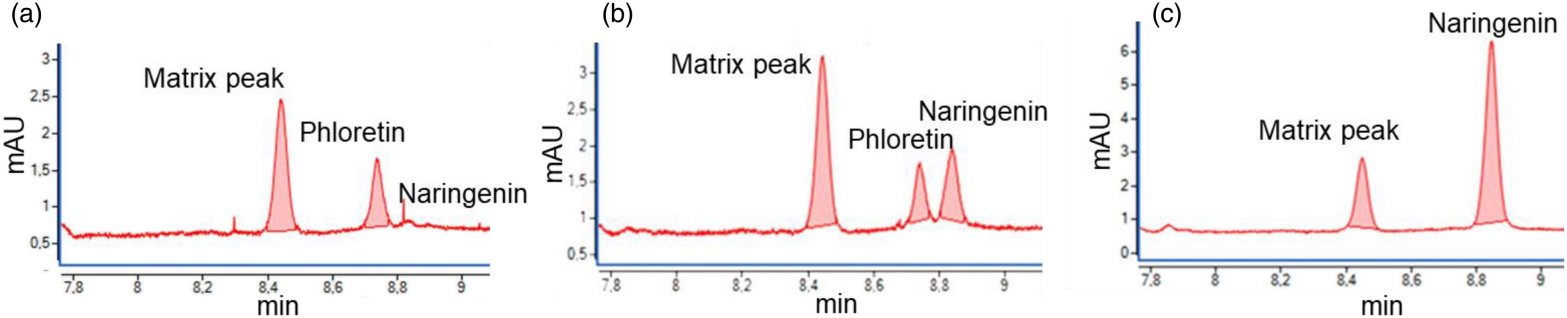
HPLC chromatograms qualitatively demonstrating the functional activity of the HDR proteoforms. (a) Proteoform without the N‐terminal amino acids 2–35 in front of the predicted proteolytic cleavage site (*Md*HDR‐ΔN). (b) Canonical *Md*HDR including the N‐terminus. (c) Negative control with *E. coli* harbouring the empty vector. The recombinant proteins were obtained from heterologous expression as glutathione S‐transferase (GST) tagged proteins in *E. coli* BL21(DE3) and the GST‐tags were removed with PreScission Protease. After incubation of the recombinant proteins with *p*‐coumaroyl‐CoA and NADPH, the formation of *p*‐dihydrocoumaroyl‐CoA was demonstrated by its conversion into phloretin after the addition of recombinant chalcone synthase (CHS) and malonyl‐CoA. Unconverted *p*‐coumaroyl‐CoA appears as naringenin in the HPLC chromatograms. An alternative product identification method using saponification of the reaction products was performed according to Ibdah *et al*. (2014) and is shown in Figure S3.

Our initial focus was on investigating the catalytic HDR activity of the proteoform *Md*HDR‐ΔN, lacking the N‐terminal amino acids in front of the predicted proteolytic cleavage site at position 36. Cloning of the *HDR* from leaves of ‘Golden Delicious’ led to the identification of two alleles (GenBank database accession numbers PQ463969, PQ463970), differing in three amino acid positions (179 D/E, 238 V/M and 280 H/R; positions refer to the canonical *Md*HDR). Both allels of *MdHDR‐ΔN* were inserted into the pGEX‐6P‐1 vector and heterologously expressed as glutathione *S*‐transferase (GST) tagged proteins in *E. coli* BL21(DE3). Protein yields were, however, very low, with approximately 30 μg/L medium. After cleavage of the GST‐tag, the functional activity of both recombinant proteins was demonstrated. Incubation of the *Md*HDR‐ΔN with *p*‐coumaroyl‐CoA, in the presence of NADPH led to the formation of *p*‐dihydrocoumaroyl‐CoA, which was demonstrated by the formation of *p*‐dihydrocoumaric acid after saponification of the products according to Ibdah *et al*. (2014) (Figure S3). This was further confirmed by phloretin formation through combined HDR/CHS/CHI assays, which avoids oxidation processes during long incubation times and heat treatment. (Figure 4a). Other hydroxycinnamic acid derivatives were tested as substrates. Feruloyl‐CoA and sinapoyl‐CoA were also accepted, whereas with caffeoyl‐CoA, only traces of product could be observed. The presence of the corresponding free acids (lacking the CoA) *p*‐coumaric acid, caffeic acid and ferulic acid, did not result in the formation of any product (Table S3). Furthermore, it was investigated whether the HDR can convert substrates, which were previously tested with *Md*DBR (Caliandro *et al*., 2021; Ibdah *et al*., 2014) and NCR (Yauk *et al*., 2024). *p*‐Coumaraldehyde, *p*‐hydroxybenzalacetone, 3‐methoxy‐4‐hydroxy‐benzalacetone, benzalacetone and naringenin chalcone were not accepted as substrates by HDR (Table S3). No differences in the substrate acceptance of the two alleles of *Md*HDR‐ΔN have been observed. The N terminus did not affect the catalytic activity. The recombinant canonical *Md*HDR, which include the N terminus, showed also functional activity (Figure 4b).

### Heterologous expression of HDR proteoforms in *Arabidopsis thaliana*

**Figure 5:**
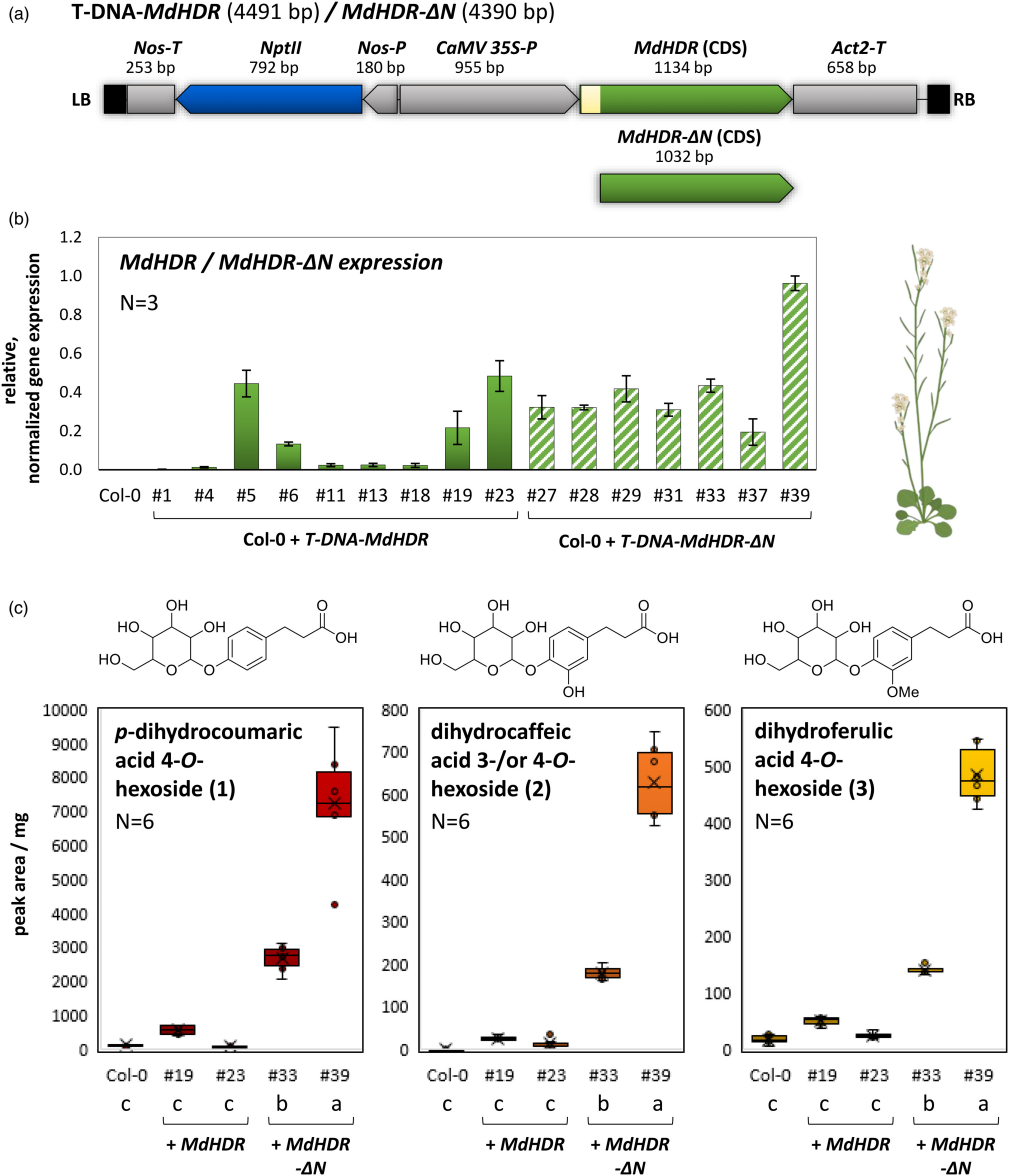
Heterologous expression of *HDR* variants in *Arabidopsis thaliana* and metabolic products identification. (a) The T‐DNA for the expression of the proteoforms *MdHDR* or *MdHDR‐∆N*, respectively, is illustrated schematically, flanked by left and right border sequences (LB and RB). The resistance cassette comprises an *NptII* resistance gene (blue) controlled by the promoter and terminator from the *nopaline synthase gene* (*Nos‐P*, *Nos‐T*). The expression of *MdHDR* or *MdHDR‐∆N* coding sequeces (CDS, green) is governed by the *Cauliflower mosaic virus* 35S promoter (*CaMV 35S‐P*) and the *Actin 2* terminator from *A. thaliana (Act2‐T)*. (b) The Col‐0 wild type and the transgenic lines were grown under greenhouse conditions for 4 weeks in three independent experiments (*N* = 3). A pooled sample from five plants, each with one rosette leaf, was collected for RNA extraction and cDNA systhesis. qPCR was conducted using a primer pair capable of detecting *MdHDR* and *MdHDR‐ΔN*. The average value of the normalized relative gene expression from the biological replicates of each experiment is depicted in a bar chart, with the error bars indicating the standard deviation. (c) Plants of Col‐0 and selected transgenic lines were grown under greenhouse conditions and six biological replicates (*N* = 6) of a pooled sample consisting of 25 leaves (five leaves from five plants) were analysed. The relative quantity of the metabolites *p*‐dihydrocoumaric acid 4‐*O*‐hexoside, dihydrocaffeic acid 3‐/or 4‐*O*‐hexoside and dihydroferulic acid 4‐*O*‐hexoside is represented as peak area per fresh weight in the box plot diagrams. The letters a, b, and c indicate the groups that were determined using Tukey's HSD (honestly significant difference) test (α = 0.05).

To assess its function *in planta*, different variants of *HDR* from apple were heterologously expressed in *A. thaliana*. Therefore, T‐DNA constructs (Figure 5a) containing the coding sequences for the expression of the canonical *Md*HDR proteoform and the N‐terminal truncated *Md*HDR‐∆N, respectively, were introduced into the wild‐type Col‐0 through *Agrobacterium*‐mediated transformation. Independent transgenic lines were established with the T‐DNA integrated homozygously at a single locus. A total of nine lines carrying the *T‐DNA‐MdHDR* and seven lines with the *T‐DNA‐MdHDR‐∆N* were further examined (Figure 5b). The presence of the T‐DNA was confirmed by PCR from genomic DNA, and the expression of the *HDR* variant (*MdHDR* and *MdHDR‐ΔN*) and *nptII* was initially verified by RT‐PCR (Figure S4). The expression of the respective *HDR* variant could be detected in all lines. To exclude any mix‐ups in the establishment process, PCR products specific to each construct were amplified from genomic DNA of two selected lines each (*MdHDR*: #19, #23; *MdHDR‐∆N*: #33, #39) and validated through Sanger sequencing. In addition, the expression levels of the respective *HDR* variants were quantified by qPCR in plant material from three independent experiments (Figure 5b). The expression levels of *MdHDR* varied among the lines, with lines #5, #6, #19 and #23 exhibiting the highest expression level. All lines carrying *T‐DNA‐MdHDR‐∆N* demonstrated noticeable *MdHDR‐∆N* expression, with line #39 displaying a level twice as high as the other *T‐DNA*‐*MdHDR‐∆N* carrying lines.

### Metabolic products formed in *HDR* transgenic lines of *A. thaliana*

To screen for putative metabolic products associated with the heterologous expression of *MdHDR* or *MdHDR‐∆N* in *A. thaliana*, hydromethanolic extracts of wild‐type Col‐0 and transgenic lines were prepared and analysed by RP‐UHPLC/ESI‐QTOFMS. Contrary to apple, the dihydrochalcones phloretin and 3‐hydroxyphloretin and their glycoconjugates were not detected in any of the analysed samples, but transgenic lines carrying the *T‐DNA‐MdHDR‐∆N* accumulated three metabolites (Figure 5c, **1**–**3**), whose analytical data were in agreement with *O*‐hexosylated dihydrocinnamic acids. Metabolites **1–3** were detected in negative ion mode via corresponding [M‐H]^−^ ions at *m/z* 327.1088 (**1**, t_R_ 2.35 min, calc. for C_15_H_19_O_8_^−^: *m/z* 327.1085), at *m/z* 343.1031 (**2**, t_R_ 2.30 min, calc. for C_15_H_19_O_9_^−^: *m*/*z* 343.1035) and at *m/z* 357.1187 (**3**, t_R_ 2.60 min, calc. for C_16_H_21_O_8_^−^: *m/z* 357.1191). Collision‐induced dissociation (CID) mass spectra obtained from [M‐H]^−^ ions of **1**–**3** at a collision energy of 10 V showed prominent Y_0_^−^ ions by loss of anhydrohexose (162.053 amu, C_6_H_10_O_5_) (Figures S5–S7). At a collision energy of 30 V, [M‐H]^−^ ions of **1**–**3** additionally formed characteristic fragment ions such as [Y_0_‐CO_2_]^−^, [Y_0_‐C_3_H_4_O_2_]^−^ and [C_2_H_3_O_2_]^−^ which are also observable in the CID mass spectra obtained from [M‐H]^−^ ions of authentic *p*‐dihydrocoumaric acid, dihydrocaffeic acid and dihydroferulic acid. Hence, metabolites **1–3** were annotated as *O*‐hexosides of *p*‐dihydrocoumaric acid, dihydrocaffeic acid and dihydroferulic acid. Metabolites **1–3** were relatively quantified in wild‐type Col‐0 and nine transgenic lines containing *MdHDR* and seven lines with *MdHDR‐∆N* (Figure S8) in samples of three biological replicates (*N* = 3) each. In all transgenic lines with *T‐DNA MdHDR‐∆N*, increased levels of *O*‐hexosylated *p*‐dihydrocoumaric acid, dihydrocaffeic acid and dihydroferulic acid could be detected compared to the wild‐type control with the highest induction in line #39 (22‐, 20‐ and 12‐fold, respectively). In this series of measurements, however, only a slight induction of the respective metabolite levels could be measured in only a few lines containing *T‐DNA MdHDR* (about 1.4‐ to 1.8‐fold induction calculated from the mean quantities in #5, #19 and #23) (Figure S8). To confirm the results, a second experiment (Table 1, Figure 5c) with more biological replicates was performed on selected transgenic lines (*MdHDR*: #19, #23; *MdHDR‐∆N*: #33, #39) in which a significant gene expression of the transgenic construct was determined before (Figure 5b). Both selected lines containing *MdHDR‐∆N* had a significantly elevated level of the quantified metabolites **1–3**. These lines exhibited a 24‐ or 64‐fold induction of *p*‐dihydrocoumaric acid *O*‐hexoside compared to the control. The average quantities of metabolites **1–3** correlate with the mean expression levels of the *MdHDR‐∆N* construct (correlation coefficients 0.994, 0.983, 0.978, respectively) in these lines. Regarding the selected lines containing the *MdHDR*, only line #19 exhibited significantly elevated quantities of metabolites **1–3**, with a lesser induction factor compared to the lines containing *MdHDR‐∆N* (Table 1, Figure 5c). No correlation between the gene expression levels and the metabolite abundances could be seen for these lines. (, ).

**Table 1:** Dihydrocinnamic acid derivatives in selected transgenic *A. thaliana* lines. Metabolite abundances are given as average fresh weight normalized peak areas [area units/mg] (*N* = 6, Figure 5c). The values of the respective transgenic line were compared to those of the control Col‐0 using a two‐sided *t*‐test, and the *P*‐value at α = 0.05 is reported. The induction factor (IF) quantifies the extent to which average metabolite abundance are induced in the transgenic lines compared to the control. n.a., not applicable

Hexosides of		*p*‐dihydrocoumaric acid			Dihydrocaffeic acid			Dihydroferulic acid		
		Average abundance	*P*	IF	Average abundnance	*P*	IF	Average abundance	*P*	IF
Col‐0		112.7			0.0			16.7		
Col‐0 + *T‐DNA‐MdHDR*	#19	564.5	0.0004	5.0	24.3	0.0012	n.a.	50.5	0.0000	3.0
	#23	90.7	0.1781	0.8	14.2	0.0336	n.a.	24.7	0.0842	1.5
Col‐0 + *T‐DNA‐MdHDR‐∆N*	#33	2664.1	0.0000	23.6	180.5	0.0000	n.a.	140.4	0.0000	8.4
	#39	7237.0	0.0002	64.2	627.8	0.0000	n.a.	484.7	0.0000	29.0

### Sequence analysis and phylogenetic studies of *Md*HDR homologues

The presence of the N‐terminal sequences in *MdHDR* seems to affect the ability of the formation of glycosylated dihydrocinnamic acids in *A. thaliana*. As predicted by MitoFates, the N terminus of the canonical *Md*HDR proteoform encodes for a TP (Figure 3b,c), that potentially targets the forming protein into the mitochondrion before it is finally removed by proteolytic cleavage (Fukasawa *et al*., 2015). The results suggest the protein lacking the N‐terminal TP is effectively involved in the formation of dihydrocinnamic acid derivatives in *A. thaliana*.

**Figure 6:**
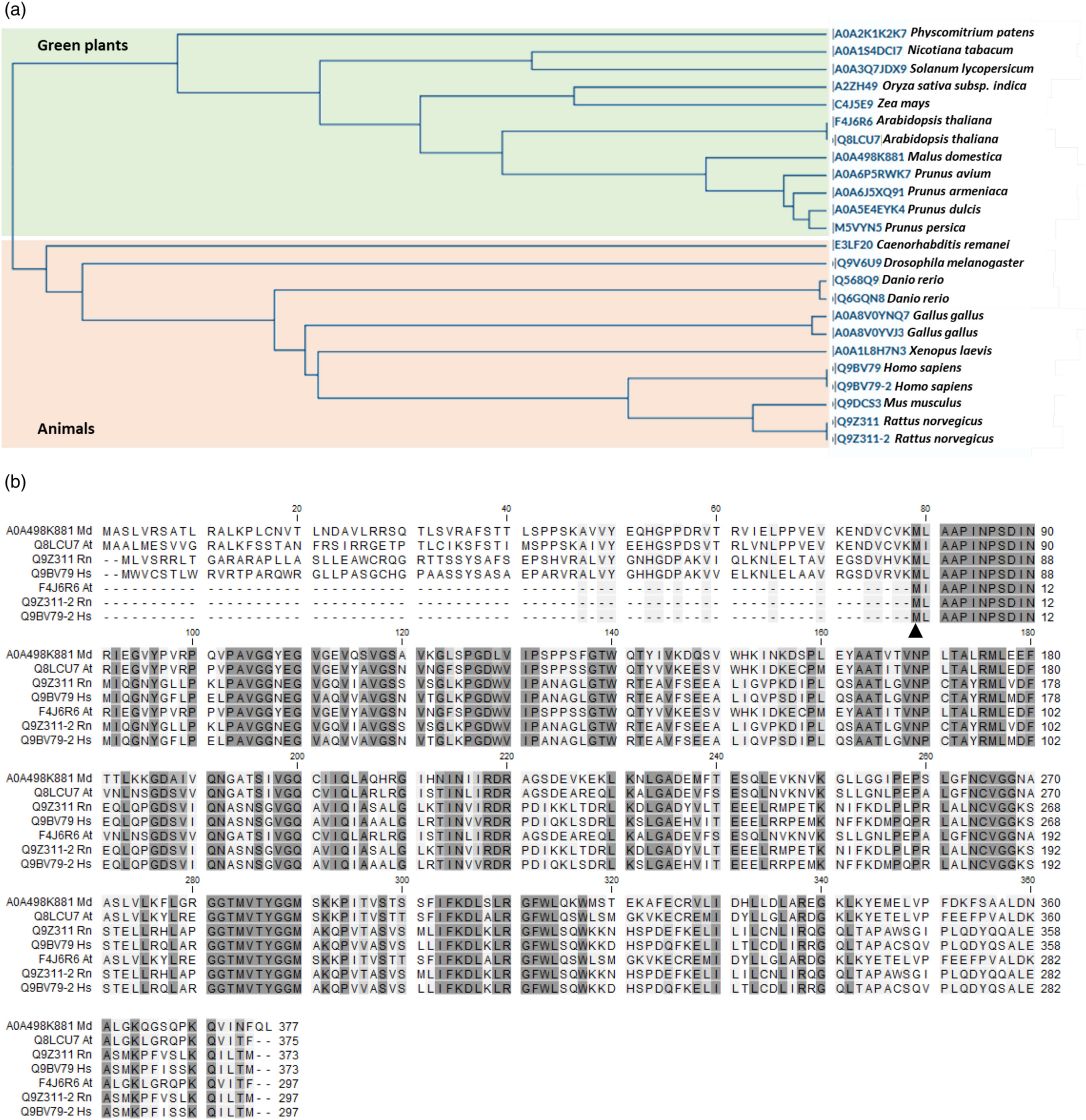
Phylogenetic tree and alignment of the *Md*HDR homologues from different species. (a) BLAST search was performed on the UniProt database (last date of access, 17.03.2025). Selected hits from several plant and animal species were used for an alignment to construct the phylogenetic tree on the Uniprot platform (clustalo program version 1.2.4.). Among 1000 significant BLAST hits, a total of 344 were identified within the clade of green plants (*Viridiplantae*), exhibiting 62%–95% amino acid identity to *Md*HDR and distributed across both monocotyledonous and dicotyledonous species. The majority of hits (609) were assigned to Metazoa, sharing 41%–53% amino acid identity. Additionally, nine hits were found in fungi, with an amino acid identity ranging from 41% to 45% (not shown). (b) Canonical proteoforms as well as proteofoms lacking the N termius from *Homo sapiens* (Hs), *Rattus norvegicus* (Rn) and *Arabidopsis thaliana* (At) are aligned to the canonical sequence of *Md*HDR (UniProt A0A498K881). The degree of conservation is represented by the grey background. The position of the alternate methionine start of the predicted cytosolic proteoforms lies within a very highly conserved region and is indicated by a triangle.

A BLAST search in the UniProt database reveals that *Md*HDR homologues are widely conserved among eukaryotes, as further illustrated by the phylogenetic tree (Figure 6a). Furthermore, it led to the identification of different homologous proteoforms (Figure 6b) that derive from a single gene in *Homo sapiens* (Q9BV79 and Q9BV79‐2 from *MECR*), *Rattus norvegicus* (Q9Z311 and Q9Z311‐2 from *MECR*) and *Arabidopsis thaliana* (Q8LCU7 and F4J6R6 from At3g45770). The human proteoform Q9BV79 is described as enoyl‐(acyl‐carrier‐protein) reductase, sharing 44.4% sequence identity with *Md*HDR and containing a TP that directs the enzyme to the mitochondria. The second proteoform Q9BV79–2 is predicted to be truncated at the N terminus (lacking 76 amino acids including the TP), thus being localized in the nucleus and cytoplasm. The latter proteoform arises from alternative splicing events and contains the first intron. This results in a frame shift of the respective coding sequences. Therefore, an alternative start codon downstream of the start codon from the canonical proteoform is used, leading to the formation of an N‐terminal truncated protein lacking the mitochondrial TP and 30 amino acids. The prediction of the human proteoform, probably located in the cytosol, is based on the evidenced alternative splicing of the enzyme homologue Q9Z311 from *R. norvegicus* (Kim *et al*., 2014).

### Screening for *HDR* transcript variants formed by alternative splicing

Due to the high sequence similarity of the HDR homologues from human, rat and apple, and in particular a conservation of the alternative start codon (Figure 6b), it was assumed that the alternative processing of the pre‐mRNA on the level of transcription or translation is also likely in the case of the apple *HDR*. To screen the apple transcriptome for alternative splice variants of the *HDR*, PacBio Iso‐Seq was performed to sequence full‐length cDNA generated from RNA obtained from leaves of ‘Gala’. In total, 18 of the obtained reads could be mapped to the locus of MD03G1107400 (Figure S9). All of them start within a very narrow interval indicating the same transcription start positions. None of these transcripts represents a splicing variant, which was expected from the comparative sequence analysis. However, our results indicate the existence of *HDR* splicing variants, revealing an alternative intron in the 3′ region. In 13 out of 18 cases, the identified transcript sequences align with the existing annotation, whereas five sequences contain an additional intron that shortens the 3′ UTR. Since the number of reads for *HDR* transcripts in the sequencing approach was low, the detection limit for splice variants with very low abundance may not have been reached. Therefore, an additional targeted enrichment sequencing approach was performed. The enrichment of *HDR* transcript sequences was achieved through nested PCRs from cDNA (Figure S10). Primers spanning the full‐length transcript, that bind to the corresponding 5′ UTR and poly‐A tail regions of the mRNA, were employed to amplify the largest possible region of the transcript. PCR products were sequenced using PacBio long‐read technology and a total of 110.979 *HDR* transcript sequences were obtained. Consistent with the initial sequencing approach, several alternative transcript variants were identified (Figure S9). However, no transcript, which was comparable to the cytosolic proteoform derived from the human homologue was found. Hence, neither of the two sequencing approaches provided evidence for a cytosolic proteoform of the *HDR* at the transcript level.

### Prediction of a cytosolic proteoform of HDR formed by alternative translation initiation

Alignment of the various enoyl‐CoA reductase homologues (Figure 6b) reveals a strong conservation in the region surrounding the alternative methionine start. To test for the presence of alternative translation initiation sites (TIS), machine learning software was used that allows the prediction of ATG and near‐cognate codon initiation sites based on a Kozak similarity score algorithm (Gleason *et al*., 2022). The start codon used for translation of the canonical *Md*HDR proteoform at mRNA position 79 was predicted with a Kozak similarity score of 0.79 (Table S4). An alternative ATG that is in frame with the canonical start codon, was predicted at position 313 with a similarity score of 0.66. If this codon is utilized for TIS, it would lead to the formation of an N‐terminal truncated proteoform, lacking the first 79 N‐terminal amino acids including the mitochondrial TP. Consequently, this proteoform would probably be located in the cytosol. Furthermore, the position of this alternative TIS corresponds to the position of the alternative ATG used in the cytosolic proteoforms. HDR activity assays performed with recombinant protein showed that the predicted cytosolic HDR proteoform exhibits catalytic activity (Figure S11).

## Discussion

Identification of the key reaction in dihydrochalcone formation, the reduction in the unsaturated C3‐bridge (Figure 1), has long been searched for. The purification approach for the isolation of a HDR used in this study started from young terminal leaves, since apple trees show a decline in phloridzin and phloretin concentration during the annual cycle (Hunter and Hull, 1993), and concurrently, most enzymes related to their formation showed the highest activity when extracted from young leaves (Kuhn, 2003). In addition, it appeared more favourable to use young leaf material, as the increasing accumulation of polyphenols during leaf growth was more likely to interfere with the isolation process of the candidate protein. The strong dependency of HDR activity on the amount of detergent during the protein extraction indicates a high binding affinity of the enzyme to hydrophobic substances of the plant material pellet. The enzyme was apparently only released into solution if very high detergent concentrations led to a saturation of the hydrophobic surfaces of the residual insoluble plant material.

One of the challenges in the characterization of HDR candidate enzymes is the lack of a direct detection of *p*‐dihydrocoumaroyl‐CoA in enzyme assays (Gosch *et al*., 2009). The current state of the art requires the processing of the formed product, either to chalcones through a combined HDR/CHS/CHI assay, or into the free acid after CoA removal via thermal treatment under alkaline conditions. Both strategies are reliable for the demonstration of functional HDR activity; however, they are less suitable for substrate specificity studies. This is because the results can be biased by the substrate specificity of the CHS, which preferentially utilizes *p*‐coumaroyl‐CoA esters as substrates, or by divergent sensitivities to oxidation and heating under alkaline conditions. In this study, the products were identified during the purification process after thermal treatment in alkaline conditions. The approach resulted in the isolation of a single enzyme, which rapidly catalyses the conversion of *p*‐coumaroyl‐CoA to *p*‐dihydrocoumaroyl‐CoA, even at low substrate concentrations. Tryptic digestion and subsequent LC‐ESI‐MS/MS analysis identified the purified enzyme with a high sequence coverage as ‘probable *trans‐2*‐enoyl‐CoA reductase, mitochondrial isoform X2 [*Malus domestica*]’ (XP_008358101) (Figure 2a). Homologous enzymes from mammals were shown to catalyse a *trans‐2‐*enoyl‐CoA reduction in fatty acid elongation (Kim *et al*., 2014; Massengo‐Tiassé and Cronan, 2009; Miinalainen *et al*., 2003), which is similar to the reduction of *p*‐coumaroyl‐CoA. In plants, fatty acid metabolism primarily takes place in the chloroplasts and peroxisomes, and bears greater structural and organizational similarity to bacterial and mitochondrial fatty acid metabolism (fatty acid synthase II).

Expression of *MdHDR* was detected in all examined tissues, although expression levels in leaves were generally higher (Figure 3). Importantly, expression levels in young leaves were approximately double compared to older leaf material, which concurs with the expectations based upon the observed higher phloridzin and phloretin concentrations in young leaves. There were noticeable variations in expression levels between the cultivars **‘**Braeburn**’**, **‘**Gala**’**, **‘**Pinova**’** and **‘**Topaz**’**, with **‘**Pinova**’** generally expressing higher levels and **‘**Gala**’** tending toward the bottom of the group, across all tissue types. In line with our qPCR analyses and transcript sequencing results, expression data from the PPGR database (Yang *et al*., 2024) indicate that *MdHDR* is expressed in all examined tissues, including roots and fruits, although its overall transcript level is relatively low (Figure S12).

The obtained amounts of HDR from apple leaves were not sufficient for a more detailed enzyme characterization. Therefore, the studies were continued with recombinant protein obtained by heterologous expression in *E. coli*. Cloning of the cDNA interestingly resulted in the isolation of two HDR alleles that differ in three amino acid positions (179 D/E, 238 V/M and 280 H/R). Both alleles were heterologously expressed from cDNA clones as full‐length proteoforms (*Md*HDR) as suggested by the annotated gene model and as truncated proteoforms lacking the putative mitochondrial TP (*Md*HDR‐∆N). Despite the low protein yields, all four proteoforms were functionally active and catalysed the conversion of *p*‐coumaroyl‐CoA to *p*‐dihydrocoumaroyl‐CoA. Feruloyl‐CoA was also accepted as a substrate. When caffeoyl‐CoA was used as a substrate, only traces of product were observed, which could be due to the higher sensitivity of dihydrocaffeic acid esters for oxidation during CoA removal. The free acids were not converted, confirming that phloretin biosynthesis does not start at an earlier stage than the CoA esters. In general, *Md*HDR accepted few of the broad range of tested substrates. Kinetic studies could not be performed because of the low yield of recombinant protein obtained. More importantly, the need for combined assays or saponification of CoA‐esters would have biased the results due to either the substrate specificity of CHS/CHI or by different oxidation sensitivity during saponification, allowing no stringent conclusions on substrate specificity. The efficient conversion of *p‐*coumaroyl‐CoA and the non‐acceptance of *p*‐coumaraldehyde particularly distinguishes *Md*HDR from the previously reported candidate enzyme *Md*DBR (Caliandro *et al*., 2021; Ibdah *et al*., 2014) and underpins its possible involvement in dihydrochalcone biosynthesis.

To investigate the functional activity *in planta*, the *HDR* was further expressed in transgenic *A. thaliana* plants, as *Md*HDR or *Md*HDR‐∆N. Elevated levels of glycosylated dihydrocinnamic acids were detected in both lines expressing *MdHDR‐∆N* and in one line expressing *MdHDR*. The presence of phloretin or phloridzin, however, could not be detected. The dihydrohydroxycinnamic acids found in transgenic lines indicate that in *A. thaliana* hydroxycinnamic acids are not metabolized to dihydrochalcones but maybe via other pathways, leading to, for example glycosylated dihydrohydroxycinnamic acids. This is in agreement with Dare (2013), who overexpressed several candidate genes in the *A. thaliana* CHI mutant *tt5* and observed unexpected changes, particularly in small phenolic acids, but not an increased phloridzin accumulation. In the transgenic lines presented in this study, the presence of hexosides of *p*‐dihydrocoumaric acid, dihydrocaffeic acid and dihydroferulic acid was unequivocally identified by mass spectrometry, thereby confirming the functional activity of HDR with and without TP *in planta*. The presence of hexosides of dihydrocaffeic acid and dihydroferulic acids in addition to *p*‐dihydrocoumaric acid does not provide a clue on the substrate specificity of the HDR, as it remains unclear whether additional hydroxyl and methyl groups are introduced before or after the reduction step. However, concentrations of the induced derivates were much higher in lines expressing *MdHDR‐∆N*, which is presumably located in the cytosol. This concurs with expectations, since flavonoid biosynthesis, and thus the hydroxycinnamic acid precursors, are located in the cytosol (Jørgensen *et al*., 2005; Obata, 2019). However, it is notable that in lines expressing the *MdHDR* construct, the concentrations of dihydrohydroxycinnamic acids were also significantly increased, if only slightly.

The presence of the N‐terminal mitochondrial TP of *Md*HDR was bioinformatics predicted with a score of 0.79. TPs initiate the transport of enzymes during and immediately after their synthesis to their subcellular destination with subsequent removal after their import (Owji *et al*., 2018). Interestingly, the previously identified genes encoding ENR‐3 and ENR‐5 (Dare *et al*., 2013a) also encode N‐terminal TPs, which indicate their subcellular location in plastids (analysed with MultiLoc28 (Höglund *et al*., 2006) and MitoFates (Fukasawa *et al*., 2015)). At first glance, the presence of a TP sequence in *Md*HDR, which directs it to the mitochondria, appeared to be incompatible with the fact that flavonoid biosynthesis, and probably phloridzin biosynthesis, occurs in the cytoplasm. However, the mammalian homologue of the apple HDR exists in two proteoforms, MECR (mitochondrial *trans*‐2‐enoyl‐CoA reductase) and cMECR (cytosolic MECR), one located in the mitochondria, initially carrying a TP, and involved in fatty acid synthesis, whereas the second protein is located in the cytoplasm and nucleus, and acts as coactivator of the nuclear hormone receptor PPARα (Kim *et al*., 2014). The cytoplasmic proteoform lacks the N‐terminal mitochondrial TP and arises from a transcript formed by alternative splicing of the first intron, leading to the use of a downstream located start codon. Due to the high similarity of the protein sequence and architecture of the mammalian and apple genes, it seems very likely that a similar processing event is also possible in apple. To prove the occurrence of different proteoforms of the *HDR*, PacBio long read sequencing was used, that allows the analysis of full‐length transcripts and is usually the state‐of‐the‐art technology when investigating for possible alternative splicing (Al‐Dossary *et al*., 2023). Different PacBio long read sequencing experiments indeed delivered alternative forms; however, none of them was comparable to the cytosolic proteoform described for both humans and rats. In addition to alternative splicing, however, there are other processing steps that could explain the formation of different proteoforms. One alternative transcript identified in this study possesses an alternative intron in the 3′ UTR region. Alterations in the 3′ UTR were described to influence post‐transcriptional control of gene expression including stability, translation and subcellular localization of the mRNA (Hong and Jeong, 2023). Furthermore, an alternative TIS could lead to the formation of a truncated cytosolic isoenzyme. Using bioinformatics analyses for the prediction of TIS (Gleason *et al*., 2022), a second start codon was predicted in the *HDR* transcript with a lower, yet not insignificant, probability. This alternative start codon is located 3′ of the canonical start codon and may provide an explanation for the formation of an N‐terminal truncated proteoform. The position of this start codon in the HDR corresponds to that of the alternative start codon found in mammalian cytosolic proteoforms, indicating a conservation of protein formation. In general, increasing knowledge on protein formation demonstrates the complexity of the process. As shown recently, start‐codon selection is dynamically regulated and dependent on structural features of the mRNA, which itself was shown to be modulated by immune challenges in plants (Xiang *et al*., 2023).

To test the functional activity of the predicted isoenzyme, the corresponding cDNA was heterologously expressed in *E. coli*. The recombinant protein showed functional activity, although much lower conversion rates were observed than with *MdHDR‐∆N*. Additional experiments will be necessary to further elucidate the localization of HDR in apple cells, for example through the use of reporter fusion proteins such as GFP. These studies will also help to dissect the functional spectrum of this protein, which may depend on its intracellular localization. It should also be noted that the transfer of results obtained from heterologous expression experiments in other plant organisms, such as *A. thaliana* and tobacco, is limited. This limitation is particularly relevant to the investigation of the biosynthetic pathway of dihydrochalcones. The massive synthesis of dihydrochalcones is a unique characteristic of apple, and it cannot be excluded that there are fundamental differences in the metabolism of these model plants that complicate the transfer of findings.

Very recently, Yauk *et al*. (2024) presented *NCR* genes (*NCR1a–c*), which seem to be responsible for the massive formation of dihydrochalcones in apple leaves via reduction of naringenin chalcone rather than reduction of *p*‐coumaroyl‐CoA. Since *NCR* gene expression could not be detected in apple fruit tissue (Yauk *et al*., 2024), it cannot be excluded that dihydrochalcone production in fruit is mediated by other enzyme(s), maybe via *p*‐coumaroyl‐CoA reduction. This is supported by expression data accessible via the PPGR database (Figure S12), showing that in contrast to *MdNCR1a‐c*, *MdHDR* is also expressed in fruits. The HDR‐dependent pathway could also be responsible for the low amount of dihydrochalcones in other plants (Rivière, 2016), if they are lacking these *NCR* genes. The presence of multiple, redundant pathways could also explain why apple mutants lacking phloridzin formation have not been identified to date.

These and other questions surrounding the formation of phloridzin in apple, and the genes involved, are of manifold scientific and technological interest, as more complete knowledge would enable the study of the physiological relevance of phloridzin in apple plants, which is still a puzzle.

Further, phloridzin is a valuable phytochemical with economically significant applications in nutrition, cosmetics, and medicine. It has traditionally been laboriously extracted from leaf material (Tian *et al*., 2021), while newer biotechnological approaches, such as apple cell suspension cultures, face challenges due to the recalcitrance of apples *in vitro* (Xu *et al*., 2025). However, the apple remains a key source for phloridzin production, due to its high content and still not fully understood metabolic pathways. The elucidation of the phloridzin biosynthesis presents the possibility of transferring phloridzin production to established, productive biotechnological systems, such as plant or bacterial cultures. Significantly, our results demonstrated that the heterologous expression of the truncated proteoform *Md*HDR‐∆N induces the metabolism for phloridzin formation. Similar bioconversion approaches have been suggested for the *trans*‐2‐enoyl‐CoA reductase TSC13 from *Saccharomyces cerevisiae* (Eichenberger *et al*., 2017) or the NCRs of apple (Yauk *et al*., 2024).

## Experimental procedures

### Chemicals, plant material and plant growth condition

All details are provided in the supplemental data section Material and methods.

### Enzyme assays

Two protocols were used for measuring HDR activity. The protocol described by Ibdah *et al*. (2014) requires a saponification step prior to HPLC quantification of substrates and products and was used during the enzyme purification and for assays with the recombinant protein. The latter were also tested with a combined HDR/CHS/CHI assay (Gosch *et al*., 2009) to verify that the products are indeed CoA esters, which can be further converted to dihydrochalcones. A detailed description of the procedures is presented in the supplemental data section Material and methods.

### Identification of candidate protein based on protein extractions

Detailed descriptions are available in the supplemental data section Material and methods. For protein extraction and removal of pigments, a protocol of Molitor *et al*. (2015) was adapted. Protein purification was performed by Fast Protein Liquid Chromatography (FPLC). The obtained fractions were tested for HDR activity, and the most promising ones were further processed. Molecular mass determination and protein identification were performed by SDS‐PAGE. A prominent protein band was excised and sent for tryptic digestion and HPLC–ESI–MS/MS experiments to an external company (Proteome Factory, Berlin, Germany). Identified peptide sequences were analysed and assigned. The obtained results were used for cloning the apple *HDR*.

### Cloning of *MdHDR* into pGEX‐6P‐1 and heterologous expression in *E. coli*

Apple mRNA was isolated from young leaves using the μMACS mRNA Isolation Kit (Miltenyi Biotec, Bergisch Gladbach, Germany) and cDNA was prepared using the RevertAid H Minus Reverse Transcriptase (Thermo Fisher Scientific, Vienna, Austria) with the oligo(dT) anchor primer AAGCAGTGGTATCAACGCAGAGTAC(T)_23_VN. Cloning into the pGEX‐6P‐1 (GE Healthcare, Vienna, Austria) bacterial expression vector was performed using an established standard procedure based on sticky end PCR cloning, as described previously (Gosch *et al*., 2014) and in the supplemental data section Material and methods. Primers were designed upon the corresponding NCBI database sequence XM_008359879 representing the purified HDR and are listed in Table S2. For expression, the plasmid constructs *MdHDR*:pGEX‐6P‐1, *MdHDR‐∆N*:pGEX‐6P‐1, and *MdHDR‐spl*:pGEX‐6P‐1 (a shortened HDR proteoform starting at an alternative downstream methionine (Figure 6b)) were transformed into *E. coli* strain BL21(DE3). Overexpression of the *HDR* variants as GST‐fusion proteins in *E. coli* by using plasmid pGEX‐6P‐1 was performed as described earlier (Gosch *et al*., 2014). Isolated, cleaved and purified proteins were shock frozen in liquid nitrogen and stored at −80 °C.

### GoldenBraid 2.0 cloning of plant transformation vectors

Plant transformation vectors were constructed with GoldenBraid cloning technology (https://gbcloning.upv.es) according to the manufacturer's instructions. The *CaMV 35S‐P* promoter (GoldenBraid database number GB0035) and *Tact2* terminator (GB0210) were used to create transcriptional units with *MdHDR* and *MdHDR‐ΔN*. The *HDRs* were joined with kanamycin resistance as a selection marker (pEGB:*Tnos*:*NptII*:*Pnos*, GB0226) resulting in the multigenic constructs pDGB3α1:*Tnos*:*NptII*:*Pnos*‐*P35s*:*MdHDR*:*Tact2* and pDGB3α1:*Tnos*:*NptII*:*Pnos*‐*P35s*:*MdHDR‐ΔN*:*Tact2*. The correct assembly was confirmed by sequencing (Microsynth, Vienna, Austria). The final multigenic constructs were transferred into *A. tumefaciens* strain GV3101 via heat‐shock treatment and used for overexpression experiments in *A. thaliana*.

### T‐DNA transformation and validation of transgenic *A. thaliana* lines

The T‐DNA constructs were stably transformed into Col‐0 by the *Agrobacterium*‐mediated floral dip method (Clough and Bent, 1998). Details on generation of homozygous, transgenic T3 lines are given in the supplemental data section Material and methods. For each transgenic line, the presence of transgenic elements was confirmed through PCR amplification using specific primer pairs (*NptII*: NptII_F /R, 779 bp; *MdHDR*: CaMV35S_F /BA_HDRoT_REV in T‐DNA_*MdHDR* 795 bp; in T‐DNA_*MdHDR‐ΔN* 693 bp). DNA for PCR templates was extracted using the DNeasy Plant Mini Kit (Qiagen, Hilden, Germany). To confirm the different *HDR* sequences in the transgenic lines, the respective full‐length coding sequences with flanking promoter and terminator sequences was amplified using 35S‐P‐FW_2/Actin2‐T‐REV (in T‐DNA_*MdHDR* 1496 bp; in T‐DNA_*MdHDR‐ΔN* 1394 bp), sequenced (Eurofins, Ebersberg, Germany) and compared with the reference sequence. The gene expression of the respective *HDR* variant was confirmed in each transgenic line by RT‐PCR using the primer pair HDR_all(2)_FW/REV (174 bp).

### Gene expression analysis

RNA was extracted from plant material of *A. thaliana* and *M*. × *domestica* using the InviTrap Spin Plant RNA Mini Kit (Invitek Diagnostics, Berlin, Germany), with genomic DNA contamination removed using the DNA‐free kit (Thermo Fisher Scientific, Schwerte, Germany). cDNA synthesis and quality control and the qPCR approach are described in detail in supplemental data section Material and methods. The optimal annealing temperature for each primer pair was determined through gradient PCR to ensure PCR specificity and efficiency (Table S2). The expression analysis of *MdHDR* in different tissues of several apple cultivars was performed with the target gene specific primer pairs MdHDR(ex1)‐FW/REV (T_a_: 59 °C; 106 bp) and MdHDR(ex1/2)‐FW/REV (Ta: 59 °C, 85 bp). For these PCRs, amplification of specific PCR products was confirmed by fragment length analysis with 2% TAE gel and sequencing of the PCR product after cloning them into a pCR blunt vector using the TA PCR cloning kit (Thermo Fisher Scientific, Schwerte). For the amplification of the reference genes the following primer pairs were used: RNAPOLII_MB1/MB2, EF1α_TM1/TM2, ACT7‐FW/REV, EF1b‐FW/EF1b‐FW, UBE210‐FW/REV and TUBB‐FW/REV.

For the expression analysis of the *MdHDR* gene construct in transgenic *A. thaliana* lines, the primer pair HDR_all_qPCR‐FW/REV (175 bp, T_a_: 61 °C) was used. The expression levels of the reference genes *AtACT1* (ACT1_2_FW /REV T_a_: 61 °C), *AtTUB2* (TUB2_2_FW /REV, T_a_: 64 °C) and *AtTIP41* (TIP41‐FW /REV, T_a_: 62 °C) (Cheng *et al*., 2022) were quantified and utilized for normalization purposes.

### Transcriptome analysis by PacBio long‐read sequencing

The transcriptome of ‘Gala’ was sequenced with PacBio Sequel II perfomed by GENEWIZ Germany GmbH (Leipzig, Germany). In a second experiment, transcripts of the *HDR* were enriched with PCR before sequencing. Detailed descriptions for target enrichment are available in the supplemental data section Material and methods and Figure S10. Hifi reads were mapped against the Golden Delicious DH genome (GDDH13_1‐1, https://iris.angers.inra.fr/gddh13/the‐apple‐genome‐downloads.html) using minimap2 (Version: 2.26‐r1175) (Li, 2018) with preset parameters for PacBio Hifi reads (Figure S9). The region of interest (Chr03 from 9 257 248 to 9 261 006 bp) was determined based on existing annotation and scrutinized with Integrative Genomics Viewer (IGV) (Robinson, 2017).

### Metabolite analysis in transgenic *A. thaliana* lines

Processing of plant material and preparation of extracts for metabolite analysis by RP‐UHPLC/ESI‐QTOFMS is described in detail in supplemental data section Material and methods. For relative quantification of dihydrocinnamic acid *O*‐hexosides, all study samples (extracts, blanks) were analysed in random order. To correct for systematic instrumental drift, pooled quality control (QC) samples were repeatedly analysed after nine study samples. In addition, four pooled QC samples were injected at the beginning and two at the end of the analytical batch. Target metabolites were quantified by integration of extracted ion chromatograms (m/z width 20 ppm) using the following quantifier ions: *p*‐dihydrocoumaric acid *O*‐hexoside, m/z 327.1085 [M‐H]‐, tR 2.35 min; dihydrocaffeic acid *O*‐hexoside, m/z 343.1035 [M‐H]‐, tR 2.30 min; dihydroferulic acid *O*‐hexoside, m/z 357.1191 [M‐H]‐, tR 2.60 min. The obtained peak areas of the study samples were corrected for instrumental drift using the peak areas of the pooled QC samples and a LOWESS/Spline interpolation algorithm (Tsugawa *et al*., 2014). Drift corrected peak areas were normalized by sample weight (Antonczak, 2008).

## Accession numbers

Sequence data have been submitted to the GenBank databases (accession numbers PQ463969 and PQ463970).

## Author contribution

HH, CHG, HF, KS and AS conceived the study. AS produced the plant material for protein isolation. CM, SS, LE, SM, MH, BW and CB produced the experimental data. CM, SS, CB, CHG, CS, HH, JK and AR analysed the data. CHG, HH, and SS, together with contributions from HF, CB, JK, and CS, drafted the manuscript, which was approved by all authors.

## Conflict of interest

The authors have not declared a conflict of interest.

## Supporting information


**Figure S1** Purification process for candidate enzyme(s) possessing hydroxycinnamoyl‐CoA double bond reductase activity.
**Figure S2** *MdHDR* gene expression in different apple cultivars and tissues.
**Figure S3** HPLC chromatograms qualitatively demonstrating the functional activity of the HDR proteoforms following the protocol of Ibdah *et al*. (2014).
**Figure S4** Validation of transgenic *Arabidopsis* lines.
**Figure S5** Collision‐induced dissociation (CID) mass spectra of *p*‐dihydrocoumaric acid *O*‐hexoside (a, b) and *p*‐dihydrocoumaric acid (c).
**Figure S6** Collision‐induced dissociation (CID) mass spectra of dihydrocaffeic acid *O*‐hexoside (a, b) and dihydrocaffeic acid (c).
**Figure S7** Collision‐induced dissociation (CID) mass spectra of dihydroferulic acid *O*‐hexoside (a, b) and dihydroferulic acid (c).
**Figure S8** Quantification of dihydrochalcone derivatives in transgenic *Arabidopsis* lines expressing *HDR* variants.
**Figure S9** *HDR* transcript sequences obtained from PacBio sequencing approaches.
**Figure S10** Sample preparation (target enrichment) of transcript variants of *HDR* for PacBio long read sequencing.
**Figure S11** HDR activity assays performed with the recombinant *Md*HDR‐spl proteoform.
**Figure S12** Expression pattern of *MdHDR* and *MdNCR1a‐c* in different tissues.
**Table S1** List of peptides found by LC/ESI‐MS/MS protein identification experiments of HDR.
**Table S2** Primers used in this study.
**Table S3** LC/ESI‐QTOFMS identification of tested substrates and obtained products in enzyme assays with recombinant *Md*HDR.
**Table S4** Prediction of translation initiation sites (TISs) in *MdHDR* mRNA.

## Data Availability

Raw data are available from the corresponding author upon reasonable request.
