# Supplementary material for: Identification of a hydroxycinnamoyl‐CoA double bond reductase (HDR) affirms multiple pathways for dihydrochalcone formation in apple
Source: Plant Biotechnol J. 2025 Jul 9;23(10):4522–35. doi: 10.1111/pbi.70225 (PMC12483985; doi:10.1111/pbi.70225)
Supplement: Supplementary file 1 — Figure S1 Purification process for candidate enzyme(s) possessing hydroxycinnamoyl‐CoA double bond reductase activity. Figure S2 MdHDR gene expression in different apple cultivars and tissues. Figure S3 HPLC chromatograms qualitatively demonstrating the functional activity of the HDR proteoforms following the protocol of Ibdah et al. (2014). Figure S4 Validation of transgenic Arabidopsis lines. Figure S5 Collision‐induced dissociation (CID) mass spectra of p‐dihydrocoumaric acid O‐hexoside (a, b) and p‐dihydrocoumaric acid (c). Figure S6 Collision‐induced dissociation (CID) mass spectra of dihydrocaffeic acid O‐hexoside (a, b) and dihydrocaffeic acid (c). Figure S7 Collision‐induced dissociation (CID) mass spectra of dihydroferulic acid O‐hexoside (a, b) and dihydroferulic acid (c). Figure S8 Quantification of dihydrochalcone derivatives in transgenic Arabidopsis lines expressing HDR variants. Figure S9 HDR transcript sequences obtained from PacBio sequencing approaches. Figure S10 Sample preparation (target enrichment) of transcript variants of HDR for PacBio long read sequencing. Figure S11 HDR activity assays performed with the recombinant MdHDR‐spl proteoform. Figure S12 Expression pattern of MdHDR and MdNCR1a‐c in different tissues. Table S1 List of peptides found by LC/ESI‐MS/MS protein identification experiments of HDR. Table S2 Primers used in this study. Table S3 LC/ESI‐QTOFMS identification of tested substrates and obtained products in enzyme assays with recombinant MdHDR. Table S4 Prediction of translation initiation sites (TISs) in MdHDR mRNA. [file PBI-23-4522-s001.docx]

**SUPPLEMENTAL DATA**

**Identification of a hydroxycinnamoyl-CoA double bond reductase (HDR) affirms multiple pathways for dihydrochalcone formation in apple**

Susan Schröpfer^1*^, Christian Haselmair-Gosch^2*^, Christoph Böttcher^3^, Christian Molitor^2^, Jens Keilwagen^4^, Lukas Eidenberger^2^, Silvija Marinovic^2^, Matthias Hackl^2^, Andreas Spornberger^5^, Benjamin Walliser^2^, Christopher Schlosser^2^, Karl Stich^2^, Annette Rompel^6^, Henryk Flachowsky^1^, Heidi Halbwirth^1**^

^1^Julius Kühn-Institute (JKI) - Federal Research Centre for Cultivated Plants, Institute for Breeding Research on Fruit Crops, Pillnitzer Platz 3a, D-01326, Dresden, Germany

^2^Technische Universität Wien, Institute of Chemical, Environmental and Bioscience Engineering, Getreidemarkt 9, 1060 Vienna, Austria

^3^Julius Kühn-Institute (JKI) - Federal Research Centre for Cultivated Plants, Institute for Ecological Chemistry, Plant Analysis and Stored Product Protection, Königin-Luise-Straße 19, 14195 Berlin, Germany

^4^Julius Kühn-Institute (JKI) - Federal Research Centre for Cultivated Plants, Institute for Biosafety in Plant Biotechnology, Erwin-Baur-Straße 27, 06484 Quedlinburg, Germany

^5^BOKU University, Institute of Viticulture and Pomology, Gregor-Mendel-Straße 33, 1180 Vienna, Austria

^6^Universität Wien, Fakultät für Chemie, Institut für Biophysikalische Chemie, 1090 Vienna, Austria

^**^corresponding author: Getreidemarkt 9, A-1060 Vienna, Austria; Tel. (43) 1 58801166559, Fax. (43) 1 5880117399, [heidrun.halbwirth@tuwien.ac.at](mailto:heidrun.halbwirth@tuwien.ac.at)

^*^contributed equally to the results

**Supplemental Figures and Tables**


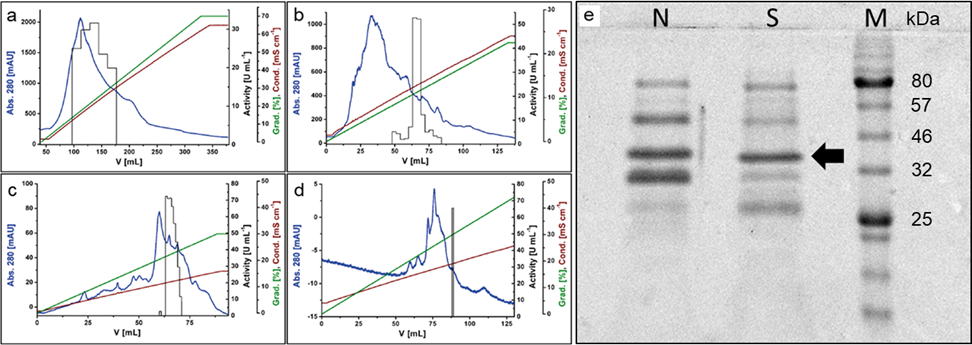


**Figure S1: Purification process for candidate enzyme(s) possessing hydroxycinnamoyl-CoA double bond reductase activity. a–d)** Example of cationic exchange chromatograms of four sequential protein purification steps. The protein was bound to the column without the presence of sodium chloride in the running buffer and was eluted with an increasing salt concentration. The gradient of the elution was 1.67 mM NaCl per column volume (CV) in b)-d). One column volume of Mono S5/50 GL had a bed volume of 1 mL. After a purification step, fractions exhibiting activity were pooled, concentrated in a Vivaspin 20 centrifugal concentrator MWCO 10 kDa (Sartorius, Göttingen, Germany) and diluted in the respective running buffer until an appropriate conductivity of ideally under 5 mS/cm was reached. **a)** SP Sepharose Fast Flow chromatography of *Malus* × *domestica* protein solution. **b)** Mono S 5/50 GL chromatography of fractions A8–B3 (96.75-160.75 mL; 64 mL pooled) from a) (MonoS1). The binding buffer was 30 mM MES pH 6.5 and the elution buffer 30 mM MES, 1 M NaCl, pH 6.5. The final concentration of 225 mM NaCl was reached after an elution volume of 135 CV. **c)** Mono S 5/50 GL chromatography of fractions IF3–IF11 (62-71 mL; 9 mL pooled) of MonoS1 (MonoS2). The binding buffer was 40 mM HEPES pH 7.2 and the elution buffer 40 mM HEPES, 500 mM NaCl, pH 7.2. The final concentration of 150 mM NaCl was reached after an elution volume of 90 CV. **d)** Mono S 5/50 GL chromatography of fractions IF3–IF11 (62-71 mL; 9 mL pooled) of MonoS2 (MonoS3). The binding buffer was 60 mM sodium acetate pH 5.6 and the elution buffer 60 mM sodium acetate, 500 mM NaCl, pH 5.6. The final concentration of 300 mM NaCl was reached after an elution volume of 180 CV. The black histograms represent measured HDR activity. The fraction volume of b)-d) was 1 mL and the activity of two to three adjacent pooled fractions was tested respectively, except for d) where only one fraction was tested each. **e)** SDS-PAGE gel of isolated and purified *M.* × *domestica* protein fractions. Fractions exhibiting the highest HDR activities were pooled and loaded onto an SDS-PAGE gel (lane S). Adjacent fractions with no HDR activity served as a negative control (lane N). Hence, proteins present in lane S but absent in lane N were regarded as candidates for the putative HDR. Lane S showed one prominent protein band, which was not present in the negative control. The protein band exhibited a molecular weight of approximately 37 kD and was identified as the most promising candidate and subjected to protein sequencing. M: Prestained Protein Standard, Broad Range 11–250 kDa (Cell Signaling Technology, Leiden, The Netherlands). The protein band marked with an arrow was excised and sequenced.


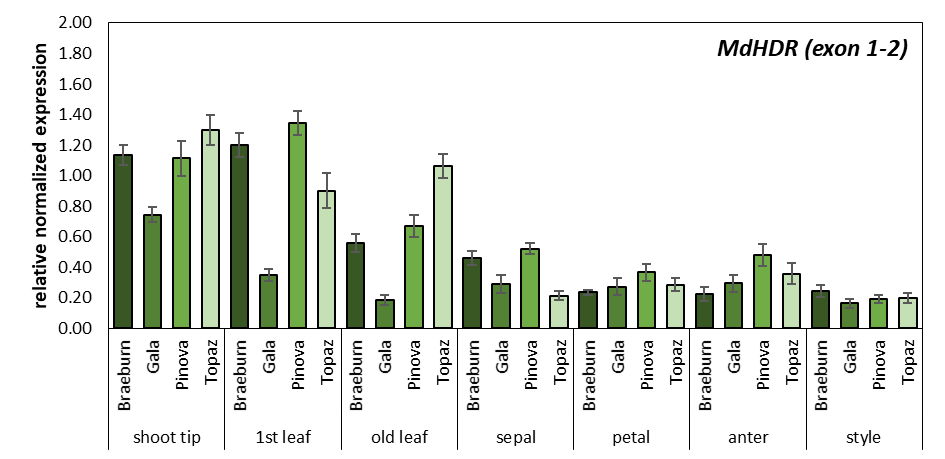


**Figure S2: *MdHDR* gene expression in different apple cultivars and tissues.** qPCR was performed with pooled samples of different tissues collected from apple trees of 'Braeburn', 'Gala', 'Pinova' and 'Topaz' using an intron spanning primer (MdHDR(ex1/2)-FW) binding in the border region of exon 1 and 2 and a reverse primer in exon 2 (MdHDR(ex1/2)-REV). The reference genes *MdEF1α*, *MdEF1β*, and *MdACT7* were used for normalization. The mean value of three technical replicates is represented as a bar and the standard deviation is given as an error bar.

**
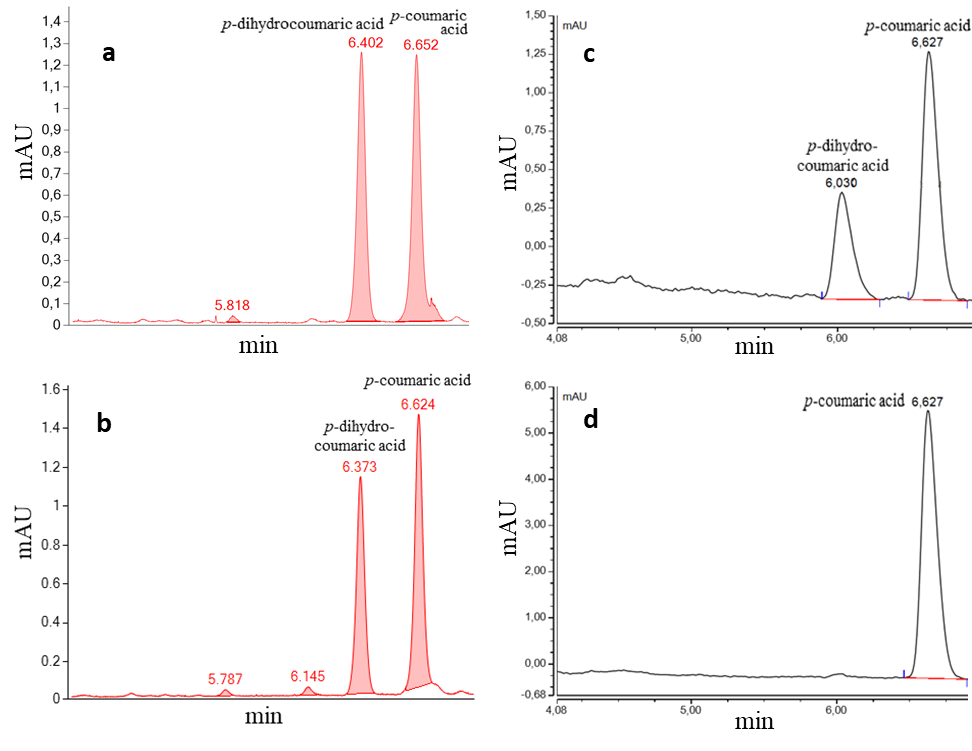
**

**Figure S3: HPLC chromatograms qualitatively demonstrating the functional activity of the HDR proteoforms following the protocol of Ibdah et al. (2014). a)** Proteoform without the N-terminal amino acids 2-35 in front of the predicted proteolytic cleavage site (*Md*HDR-ΔN). **b)** Canonical *Md*HDR including the N-terminus, both analysed by the HPLC method described in Supplemental Material and Methods section “Enzyme assays”. **c)** *Md*HDR-ΔN and **d)** the corresponding negative control with heat inactivated enzyme showing exemplarily the enzymatic activity of the HDR, both analysed with LC/MS method described in Supplemental Material and Methods section “LC/ESI-QTOFMS product identification”. Divergent graphic styles for a) and b) vs. c) and d) are the result of differing HPLC machines. The recombinant proteins were obtained from heterologous expression as glutathione *S*-transferase (GST) tagged proteins in *E. coli* BL21(DE3) and the GST-tags were removed with PreScission Protease. After incubation of the recombinant proteins with *p*-coumaroyl-CoA and NADPH, the hydroxycinnamic acid esters were saponified at alkaline conditions which allows the separation and detection of the free acids, *p*-coumaric acid and *p*-dihydrocoumaric acid. Peak areas do not provide clues on conversion rates, since *p*-dihydrocoumaric acid has a much lower absobance than *p*-coumaric acid at the detection wavelength of 276 nm.


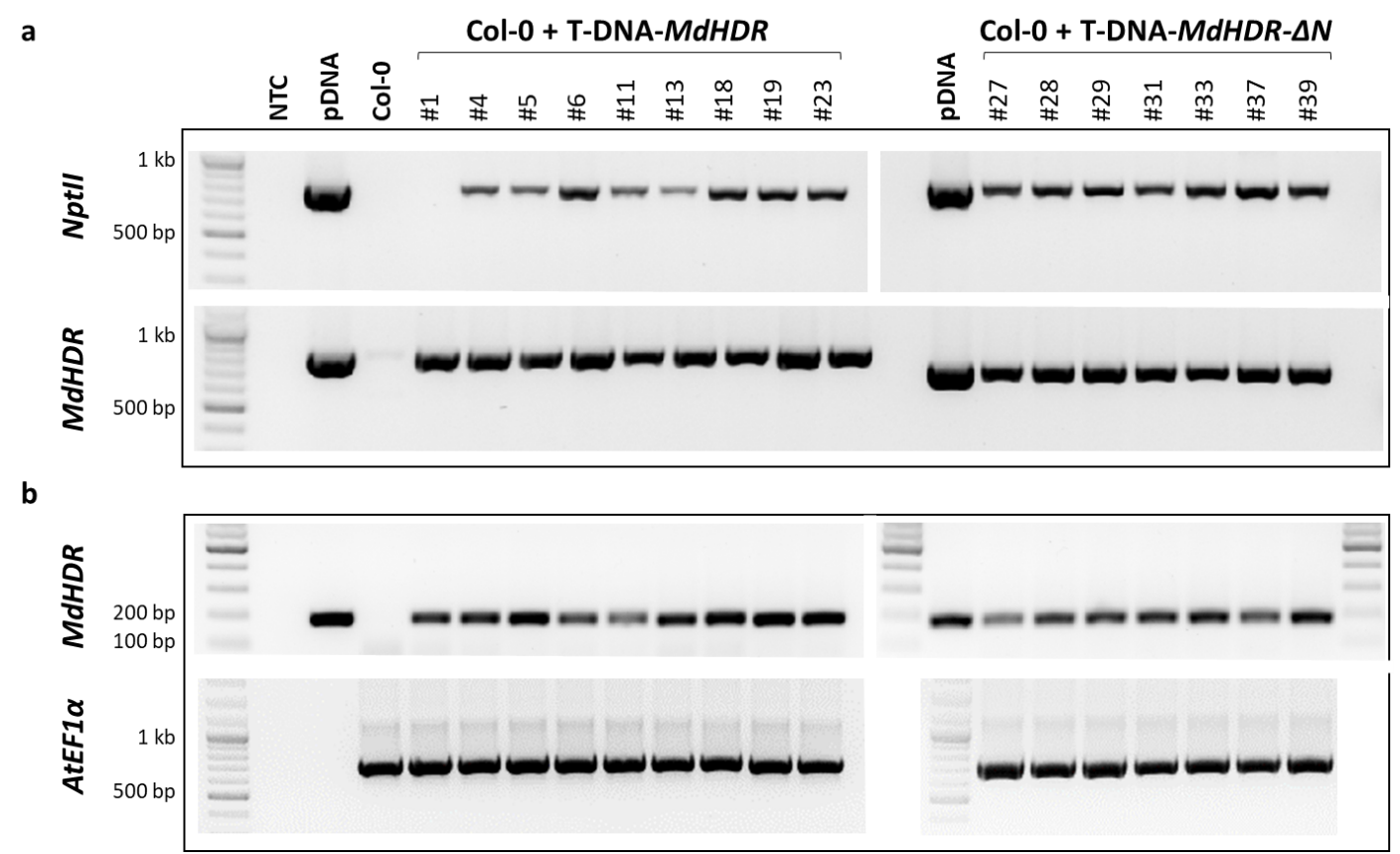


**Figure S4: Validation of transgenic *Arabidopsis* lines**. *A. thaliana* wild type Col-0 was stably transformed using *A. tumefaciens* strains containing T-DNA constructs *MdHDR* (pDGB3α1:*Tnos:NptII:Pnos-P35s:MdHDR:Tact2*) or *MdHDR-ΔN* (pDGB3α1:*Tnos:NptII:Pnos-P35s:MdHDR-ΔN:Tact2*). Independent transgenic lines (indicated by numbers) were established, each carrying the corresponding T-DNA construct homozygously at a single locus. **a)** The presence of T-DNA sequences was confirmed in each line by PCR amplification of *NptII-* and *HDR-*specific sequences from genomic DNA. **b)** RT-PCR was performed to detect the expression of the transgenic *HDR* construct in each *Arabidopsis* line using *HDR*-specific primers. The expression of the reference gene *AtEF1α* was used as an internal control. PCR-controls included No-Template-Control (NTC), wild type Col-0, and the respective plasmid DNA of the binary plasmid (pDNA).


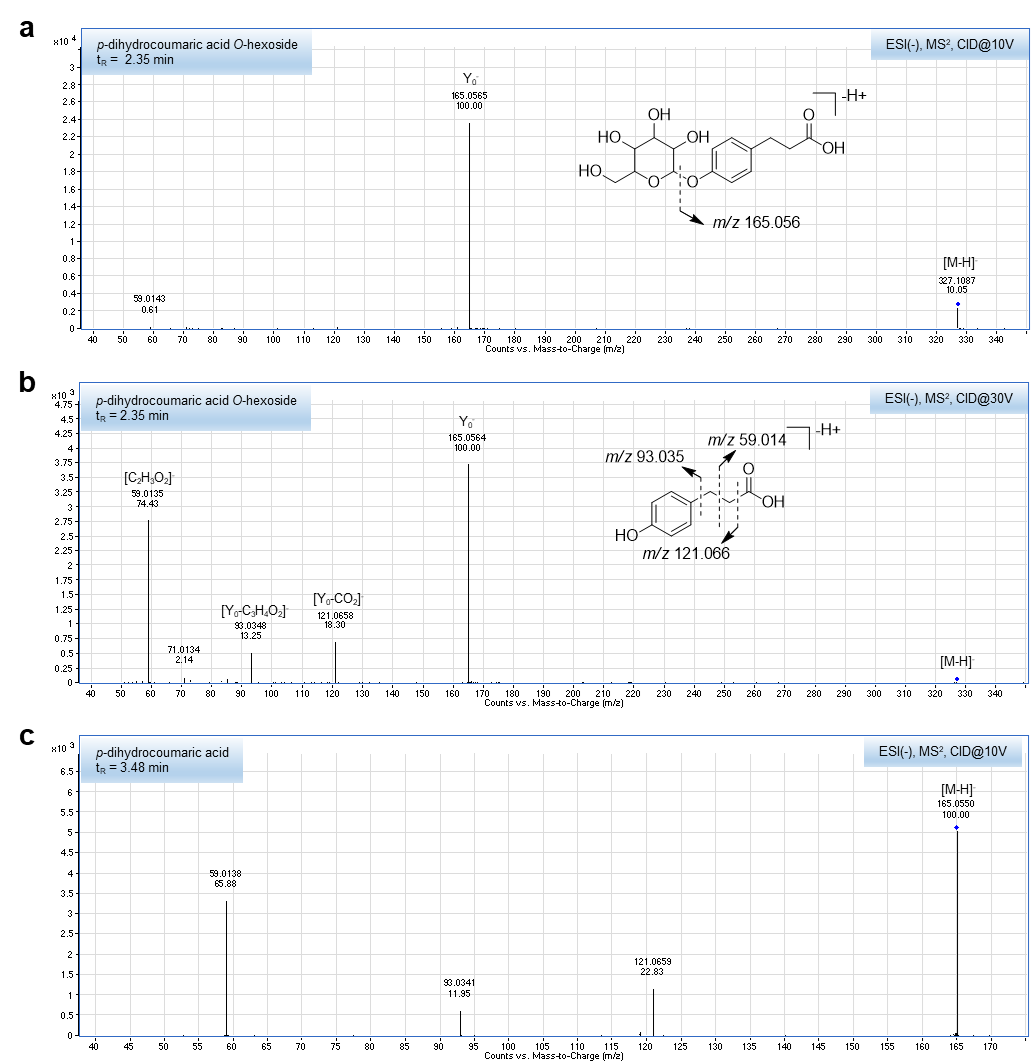


**Figure S5: Collision-induced dissociation (CID) mass spectra of *p*-dihydrocoumaric acid *O*-hexoside** **(a, b) and *p*-dihydrocoumaric acid (c).** Spectra **a** and **b** were obtained from a leaf extract of *A. thaliana* expressing *MdHDR-∆N* (line #39) using RP-UHPLC/ESI(-)-QTOFMS. Spectrum **c** was obtained from commercially available *p*-dihydrocoumaric acid using RP-UHPLC/ESI(-)-QTOFMS. Precursor ions ([M-H]-) are marked with a blue diamond.

**
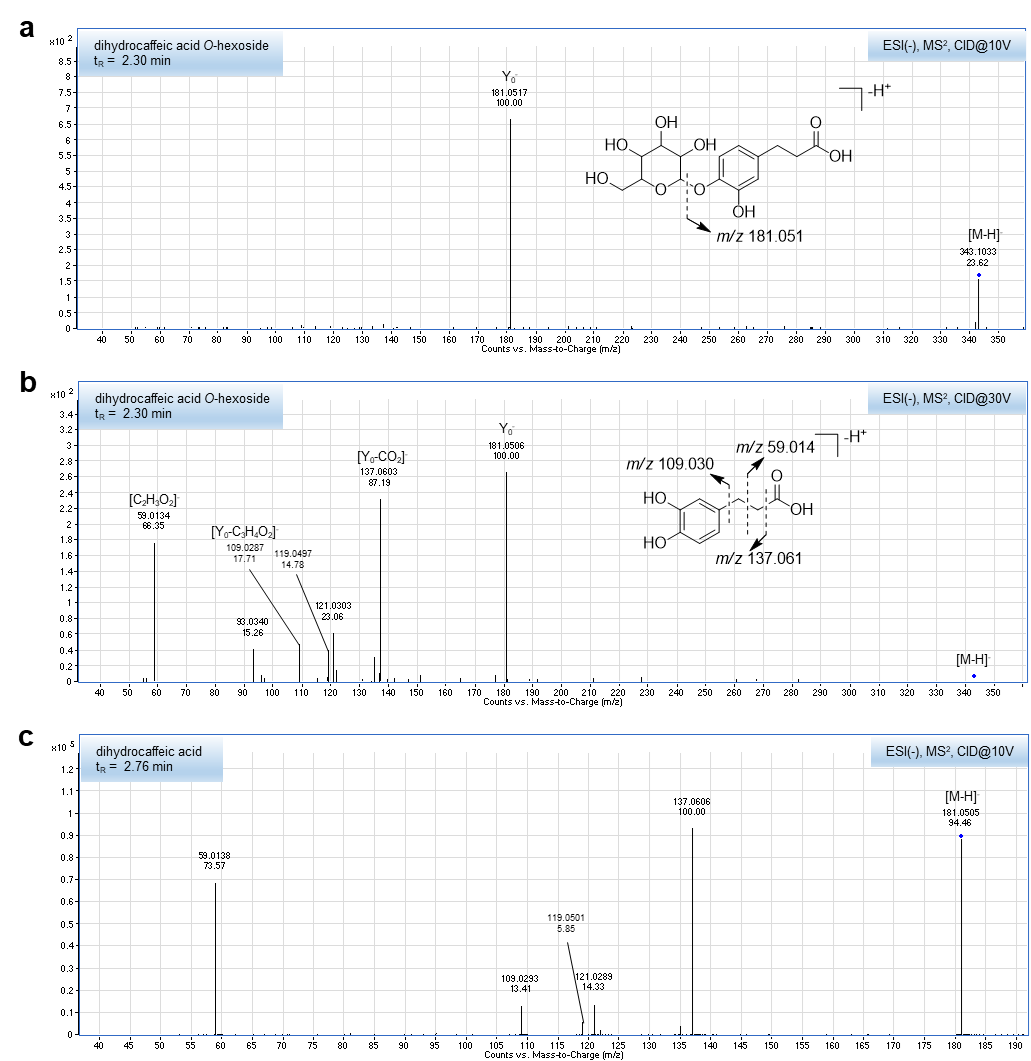
**

**Figure S6: Collision-induced dissociation (CID) mass spectra of dihydrocaffeic acid *O*-hexoside (a, b) and dihydrocaffeic acid (c).** Spectra **a** and **b** were obtained from a leaf extract of *A. thaliana* expressing *MdHDR-∆N* (line #39) using RP-UHPLC/ESI(-)-QTOFMS. Spectrum **c** was obtained from commercially available dihydrocaffeic acid using RP-UHPLC/ESI(-)-QTOFMS. Precursor ions ([M-H]-) are marked with a blue diamond.


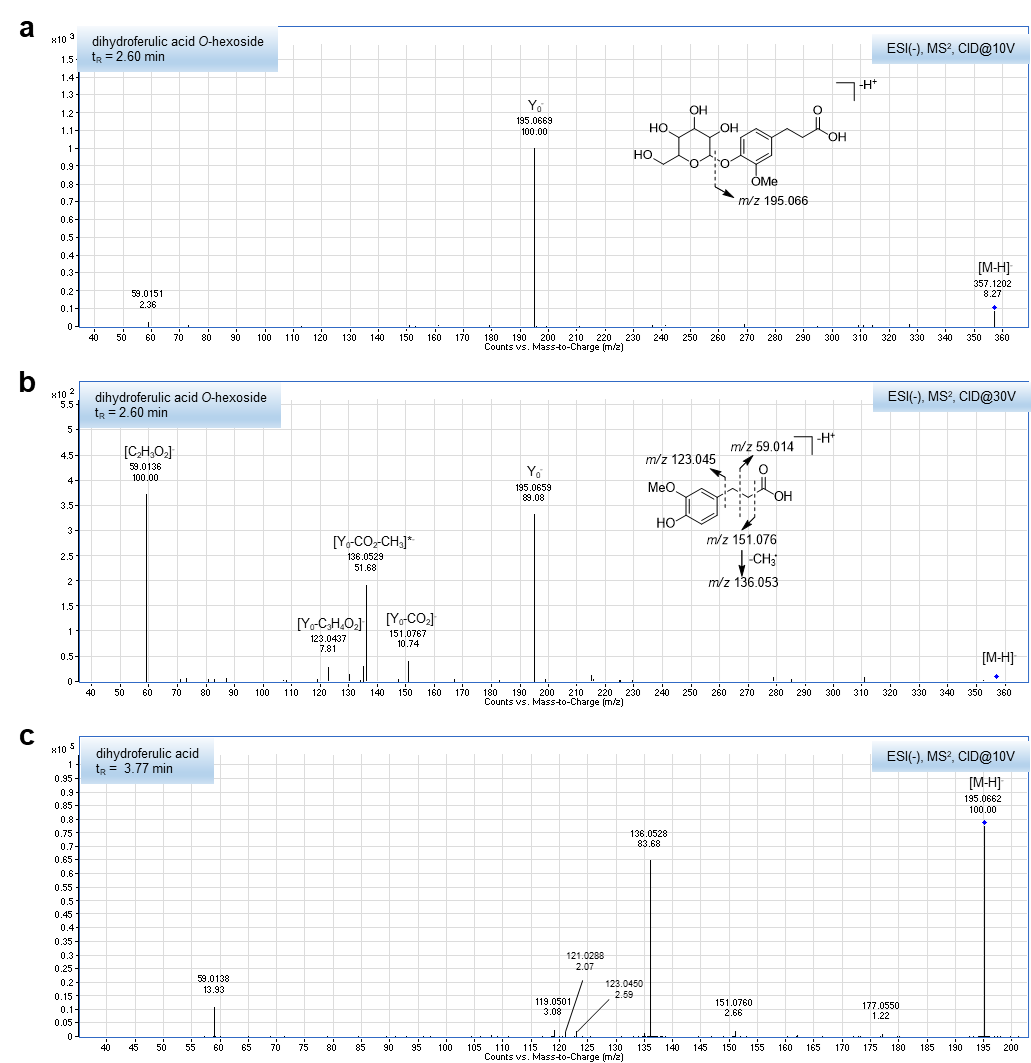


**Figure S7: Collision-induced dissociation (CID) mass spectra of dihydroferulic acid *O*-hexoside (a, b) and dihydroferulic acid (c).** Spectra **a** and **b** were obtained from a leaf extract of *A. thaliana* expressing *MdHDR-∆N* (line #39) using RP-UHPLC/ESI(-)-QTOFMS. Spectrum **c** was obtained from commercially available dihydroferulic acid using RP-UHPLC/ESI(-)-QTOFMS. Precursor ions ([M-H]-) are marked with a blue diamond.

**Figure S8: Glycosylated dihydrocinnamic acids in transgenic *Arabidopsis* lines expressing *HDR* variants. a)** Quantification of glycosylated dihydrocinnamic acids. The Col-0 wild type and the transgenic lines were grown under green house conditions for four weeks in three independent experiments. A pooled sample from five plants, each with five rosette leaves, was collected for metabolite quantification. The mean quantity of the metabolites *p*-dihydrocoumaric acid 4-*O*-hexoside (**1**), dihydrocaffeic acid 3-/or 4-*O*-hexoside (**2**) and dihydroferulic acid 4-*O*-hexoside (**3**) is represented as peak area per fresh weight in the bar charts (N=3; except Col-0, Col-0 + *T-DNA-MdHDR* #6, #19: N=2; Col-0 + *T-DNA-MdHDR* #1, #4: N=1), with the error bars indicating the standard deviation. **b)** Simplified hypothetical biosynthesis pathway leading to dihydroxycinnamic acid-*O*-hexosides in transgenic *Arabidopsis* lines expressing HDR variants.


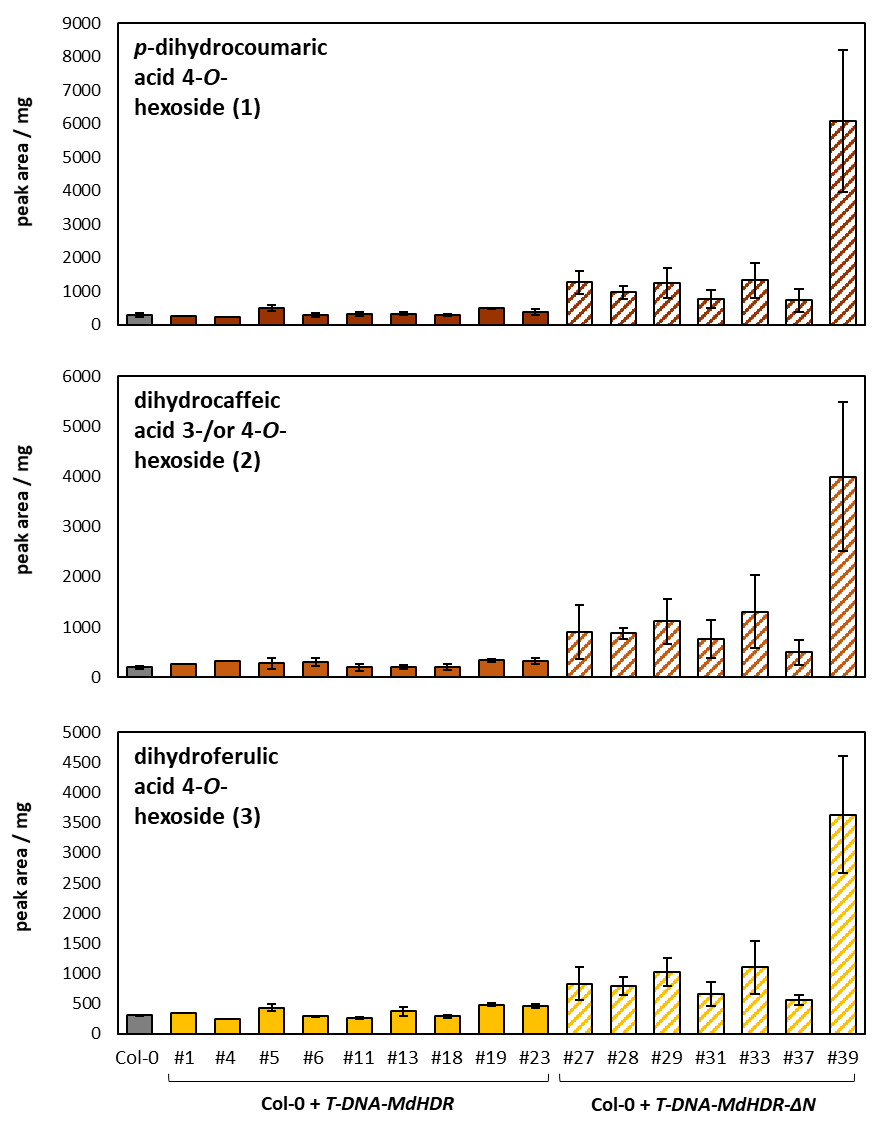


**a**

**b**


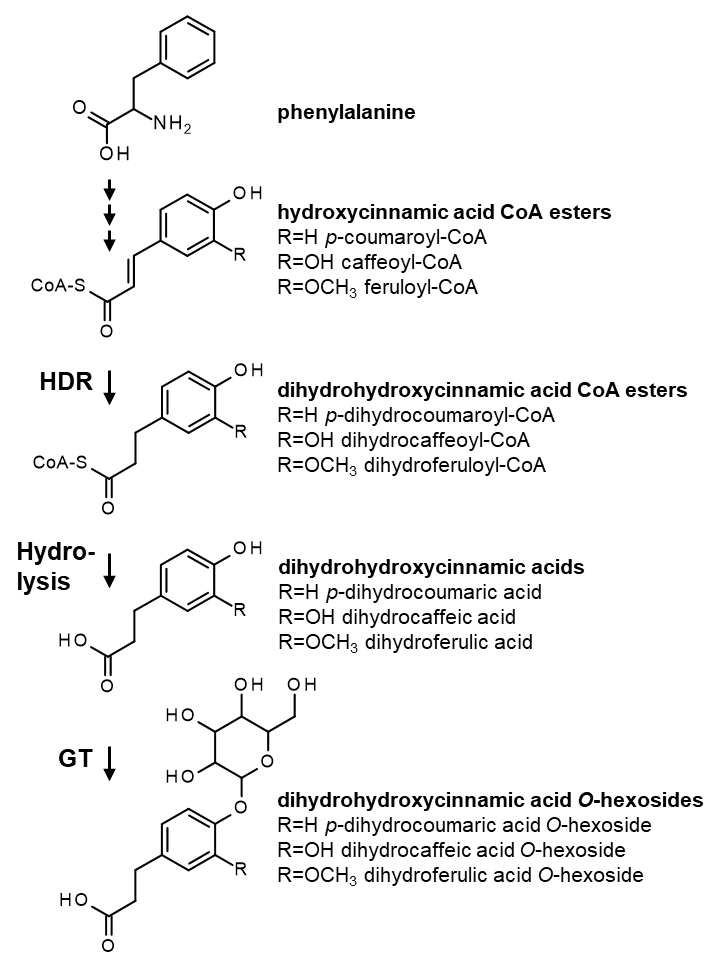


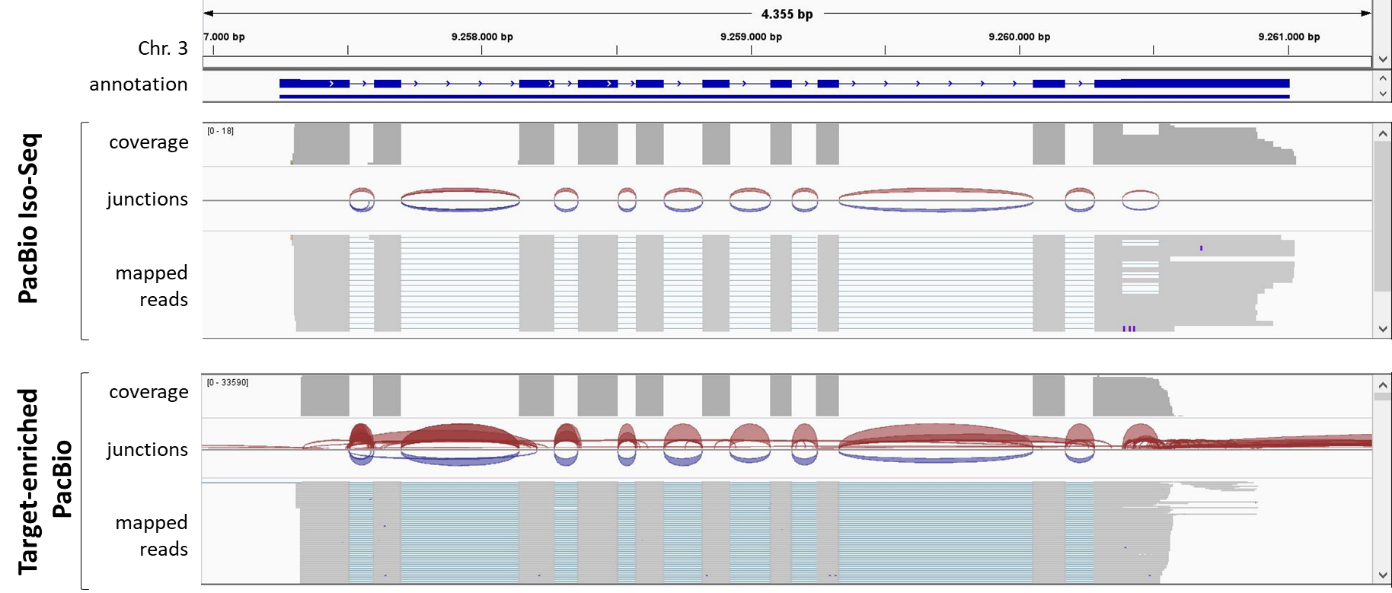


**Figure S9: *HDR* transcript sequences obtained from PacBio sequencing approaches.** The two reads obtained from PacBio Iso-Seq and the target-enriched PacBio sequencing approaches were mapped to the genomic locus MD03G1107400 on chromosome 3 of the apple reference genome GDDH13 V1.1. The schematic drawing at the top illustrates the canonical annotation of the gene, with exon sequences depicted as bold blue boxes and intron sequences represented by blue lines. The Integrative Genomics Viewer (IGV) tool (Robinson, 2017) was utilized to visualize sequence coverage, junctions, and mapped reads.


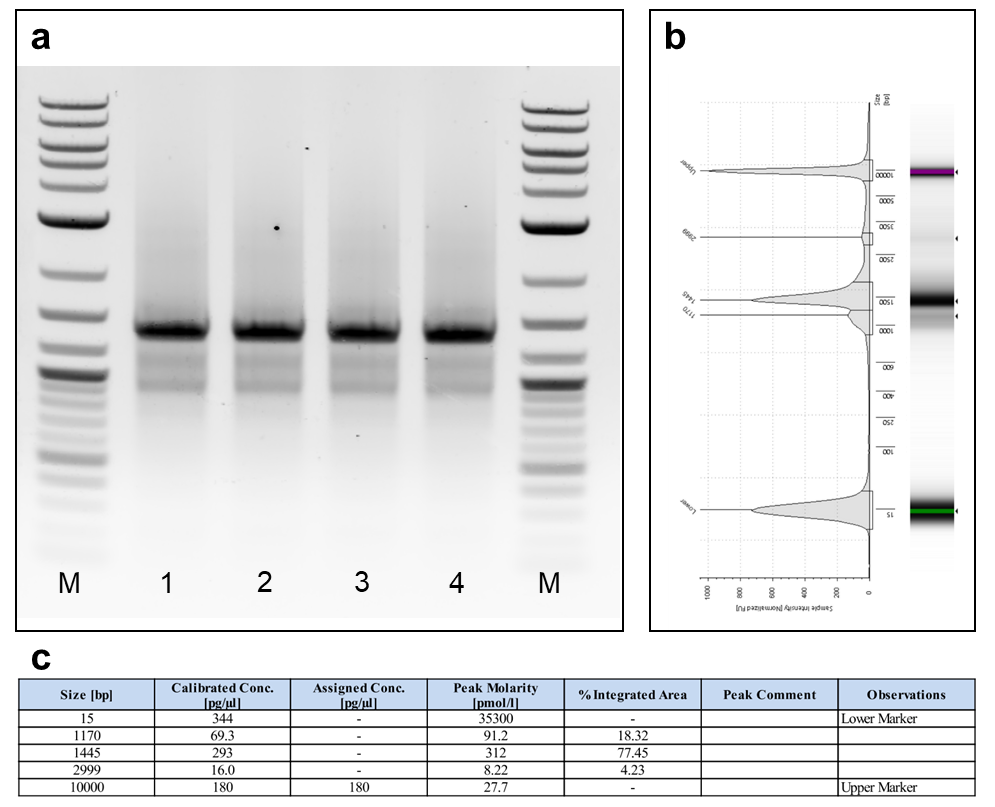


**Figure S10: Sample preparation (target enrichment) of transcript variants of *HDR* for PacBio long read sequencing.** **a)** For PCR, the primers Md_ges_nf1 and Anker_rev_1, and cDNA as a template were used, followed by a nested PCR with the primers 1_MD_ges_f and Anker_rev_1. The reverse primer Anker_rev_1 binds to the anchor sequence that was introduced during cDNA synthesis by the oligo(dT) anchor. Products between 1 and 2 kb of four PCR experiments (1–4) were cut out from a 1% agarose gel, purified, pooled and used for PacBio long read sequencing. M: DNA length marker NEB 1kb Plus DNA ladder (New England Biolabs, Frankfurt am Main, Germany). **b)** Sample quality control and **c)** peak table of pooled sample (Azenta, Leipzig, Germany). The quality parameters were: A_260/280_: 1.80, A_260/230_: 2.41; 106 ng/µL; (NanoDrop 2000; Thermo Scientific, Schwerte, Germany)

**
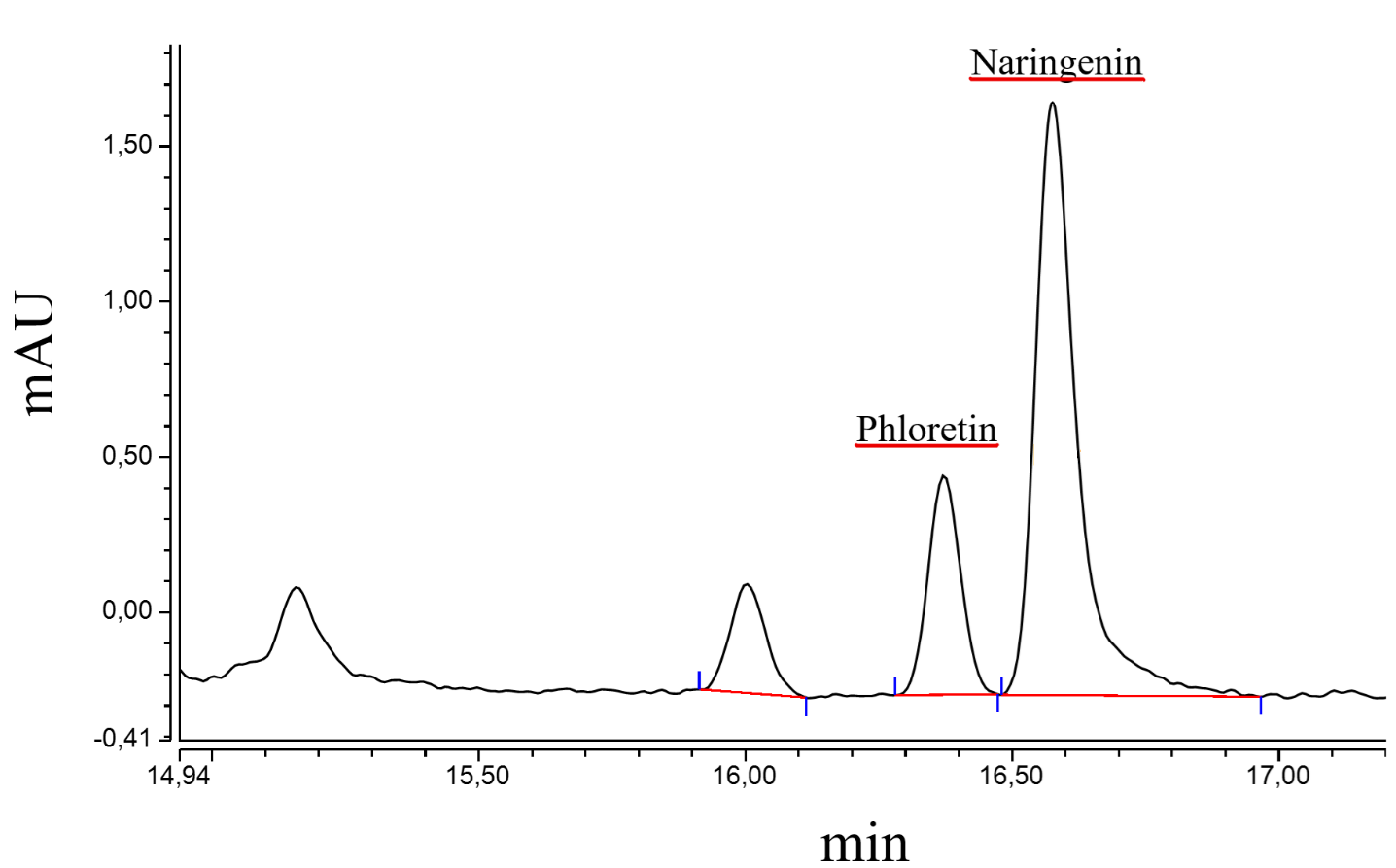
**

**Figure S11: HDR activity assays performed with the recombinant *Md*HDR-spl proteoform.** The recombinant protein was obtained from heterologous expression as glutathione *S*-transferase (GST) tagged protein in *E. coli* BL21(DE3) and the GST-tag was removed with PreScission Protease. After incubation of the recombinant protein with *p*-coumaroyl-CoA and NADPH, the formation of *p*-dihydrocoumaroyl-CoA was demonstrated by its conversion into phloretin in the HDR/CHS/CHI assay. Unconverted *p*-coumaroyl-CoA appears as naringenin in the HPLC chromatogrammes.

**
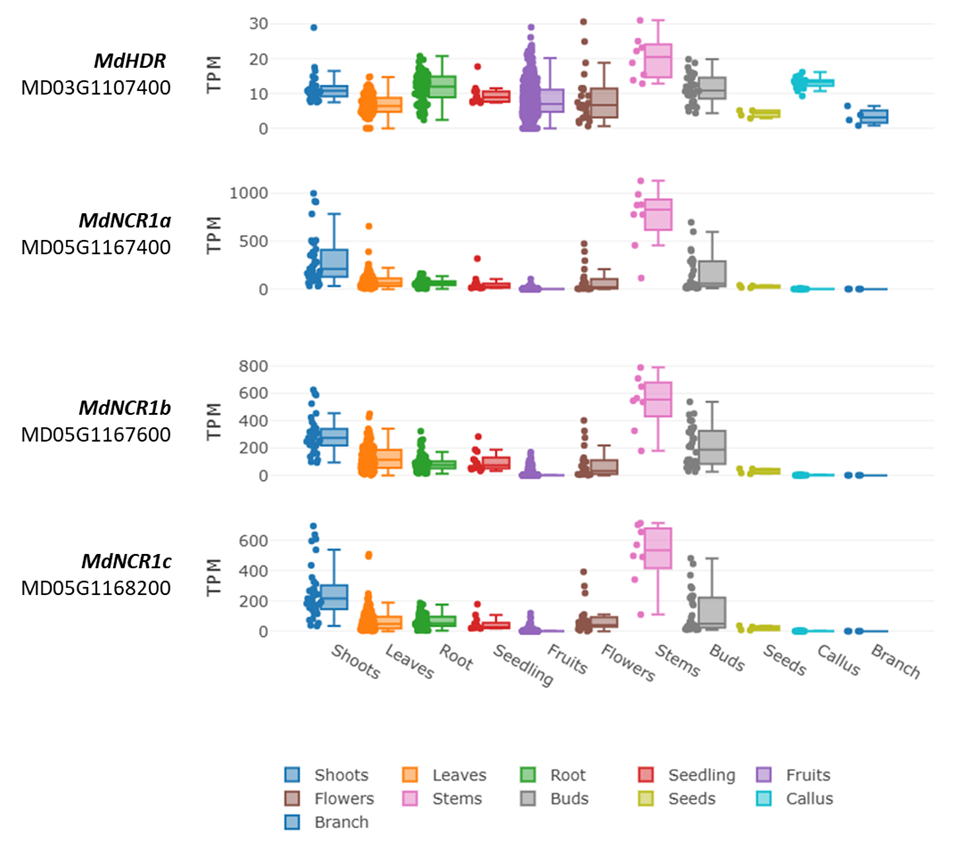
Figure S12: Expression pattern of *MdHDR* and *MdNCR1a-c* in different tissues.** For each gene, expression diagrams were obtained from the PPGR database (Resource for Perennial Plant Genomes and Regulation; last accessed on March 14, 2025) (Yang et al., 2024) and compiled for better comparison. The expression levels are represented in transcripts per million (TPM). The expression patterns of the *MdNCR1* genes are comparable to one another, with the highest expression observed in shoots, stems, and buds. In some tissues, such as fruits, seeds, callus, and branches, transcripts of *MdNCR1a-c* are nearly absent (median TPM fruit: 0,0). In leaves and roots, only a lower expression level was measured compared to shoots and stems. Overall, the level of transcripts detected for *MdHDR* is lower than that of *MdNCR1a-c*; however, it was detected in each analyzed tissue, including roots and fruits. The median level of *MdHDR* expression in fruits (median 6,96 TPM) is comparable to the expression levels measured in shoots, leaves, and roots.

Phloretin

**Table S1: List of peptides found by LC–ESI-MS/MS protein identification experiments of HDR.** Sequence coverage: 65%. Data analysis by Proteome Factory (Berlin, Germany) using Mascot (www.matrixscience.com). Start – End (according to NCBI database sequence XP_050140734.1): Location of the identified peptide within the protein database sequence. m/z value detected: m/z value (mass to charge ratio) measured by mass spectrometry. Mr detected: Experimentally determined mass. Mr calculated: Monoisotopic mass of the peptide calculated from the sequence. Mass error ppm: Relative deviation of the measured value Mr detected from the theoretical value Mr calculated per million. Match score: Value indicating how well the corresponding peaks in the tandem mass spectrometry matches the predicted spectrum (values >20 are reliable). Expect value: Statistical significance of the hit in relation to the threshold value (0.05). Peptide: Sequence of the peptide. Adjacent amino acids cut off by proteolysis with trypsin are left and right of the dots. (M), (NQ): Oxidation of methionine (M) and deamidation of asparagines (N) and glutamines (Q), which cause mass shifts.

| **Start – End** | **m/z value detected** | **Mr**  **detected** | **Mr**  **calculated** | **Mass error ppm** | **Match score** | **Expect value** | **Peptide** |
| --- | --- | --- | --- | --- | --- | --- | --- |
| 36 – 46 | 568.3026 | 1134.5907 | 1134.5921 | -1.20 | 53 | 0.0074 | R.AFSTTLSPPSK.A |
| 47 – 58 | 684.3399 | 1366.6652 | 1366.6630 | 1.66 | 75 | 4.5e-005 | K.AVVYEQHGPPDR.V |
| 62 – 71 | 561.8415 | 1121.6684 | 1121.6696 | -1.10 | 65 | 7.8e-005 | R.VIELPPVEVK.E |
| 62 – 78 | 984.0295 | 1966.0444 | 1966.0445 | -0.076 | 45 | 0.046 | R.VIELPPVEVKENDVCVK.M |
| 79 – 91 | 706.3706 | 1410.7266 | 1410.7289 | -1.64 | 70 | 0.00011 | K.MLAAPINPSDINR.I |
| 79 – 91 | 714.3684 | 1426.7222 | 1426.7238 | -1.11 | 120 | 1.2e-009 | K.MLAAPINPSDINR.I + Oxidation (M) |
| 92 – 122 | 782.6655 | 3126.6331 | 3126.6350 | -0.62 | 66 | 0.00032 | R.IEGVYPVRPQVPAVGGYEGVGEVQSVGSAVK.G |
| 92 – 122 | 782.6660 | 3126.6348 | 3126.6350 | -0.074 | 80 | 1.3e-005 | R.IEGVYPVRPQVPAVGGYEGVGEVQSVGSAVK.G |
| 154 – 175 | 1193.6541 | 2385.2936 | 2385.2903 | 1.36 | 66 | 0.00023 | K.INKDSPLEYAATVTVNPLTALR.M |
| 176 – 184 | 549.2822 | 1096.5499 | 1096.5474 | 2.29 | 63 | 0.00054 | R.MLEDFTTLK.K |
| 176 – 184 | 557.2777 | 1112.5409 | 1112.5424 | -1.29 | 52 | 0.0054 | R.MLEDFTTLK.K + Oxidation (M) |
| 176 – 185 | 621.3268 | 1240.6391 | 1240.6373 | 1.43 | 46 | 0.03 | R.MLEDFTTLKK.G + Oxidation (M) |
| 185 – 209 | 893.1436 | 2676.4089 | 2676.4130 | -1.53 | 87 | 2.3e-006 | K.KGDAIVQNGATSIVGQCIIQLAQHR.G |
| 185 – 209 | 893.4744 | 2677.4014 | 2677.3970 | 1.64 | 75 | 4.5e-005 | K.KGDAIVQNGATSIVGQCIIQLAQHR.G + Deamidated (NQ) |
| 186 – 209 | 1275.6633 | 2549.3120 | 2549.3020 | 3.92 | 111 | 1.4e-008 | K.GDAIVQNGATSIVGQCIIQLAQHR.G + Deamidated (NQ) |
| 210 – 218 | 525.3156 | 1048.6166 | 1048.6141 | 2.30 | 41 | 0.027 | R.GIHNINIIR.D |

Table is continued on next page

Table continued from previous page

| **Start – End** | **m/z value detected** | **Mr**  **detected** | **Mr**  **calculated** | **Mass error ppm** | **Match score** | **Expect value** | **Peptide** |
| --- | --- | --- | --- | --- | --- | --- | --- |

| 219 – 229 | 411.8737 | 1232.5992 | 1232.5997 | -0.41 | 47 | 0.029 | R.DRAGSDEVKEK.L |
| --- | --- | --- | --- | --- | --- | --- | --- |
| 230 – 247 | 1010.5332 | 2019.0519 | 2019.0524 | -0.26 | 115 | 5.6e-009 | K.LKNLGADEVFTESQLEVK.N |
| 277 – 292 | 821.8899 | 1641.7653 | 1641.7644 | 0.54 | 90 | 1.2e-006 | K.FLGHGGTMVTYGGMSK.K |
| 277 – 292 | 829.8885 | 1657.7625 | 1657.7593 | 1.92 | 74 | 4.3e-005 | K.FLGHGGTMVTYGGMSK.K + Oxidation (M) |
| 277 – 292 | 837.8847 | 1673.7548 | 1673.7542 | 0.38 | 59 | 0.0013 | K.FLGHGGTMVTYGGMSK.K + 2 Oxidation (M) |
| 293 – 305 | 727.9161 | 1453.8176 | 1453.8181 | -0.32 | 71 | 4.5e-005 | K.KPITVSTSSFIFK.D |
| 328 – 338 | 639.3821 | 1276.7497 | 1276.7503 | -0.45 | 82 | 1.8e-006 | R.VLIDHLLDLAR.E |
| 342 – 353 | 756.3937 | 1510.7729 | 1510.7741 | -0.83 | 77 | 2.6e-005 | K.LKYEMELVPFDK.F |
| 342 – 353 | 764.3936 | 1526.7727 | 1526.7690 | 2.42 | 89 | 1.8e-006 | K.LKYEMELVPFDK.F + Oxidation (M) |
| 344 – 353 | 635.8045 | 1269.5945 | 1269.5951 | -0.52 | 70 | 0.00011 | K.YEMELVPFDK.F |
| 344 – 353 | 643.8031 | 1285.5917 | 1285.5900 | 1.33 | 52 | 0.0065 | K.YEMELVPFDK.F + Oxidation (M) |
| 344 – 353 | 643.8046 | 1285.5947 | 1285.5900 | 3.62 | 69 | 9.9e-005 | K.YEMELVPFDK.F + Oxidation (M) |
| 354 – 364 | 553.7955 | 1105.5765 | 1105.5768 | -0.27 | 87 | 3.3e-006 | K.FSAALDNALGK.Q |
| 354 – 364 | 554.2872 | 1106.5599 | 1106.5608 | -0.79 | 81 | 1.1e-005 | K.FSAALDNALGK.Q + Deamidated (NQ) |
| 371 – 377 | 431.2453 | 860.4760 | 860.4756 | 0.45 | 49 | 0.016 | K.QVINFQL.- |

**Table S2: Primers used in this study.**

| **Name** | **Oligo sequence (5'-3')** | **qPCR efficiency [%]** | **Ta** | **Purpose** | **Gene/Reference** |
| --- | --- | --- | --- | --- | --- |
| **Primer pairs for *HDR* qPCR analysis in apple** | |  |  |  |  |
| RNAPOLII_MB1 | ATATGCCACCCCGTTCTCTACT | 100.0 | 62°C | reference gene | *MdRNAPOLII (RNA polymerase II)* |
| RNAPOLII_MB2 | CACGTTCCATTTGTCCAAACTT |  |  |  |  |
| EF1α_TM1 | TCAAGCGTGGGTACGTTGCTTC | 97.6 | 66°C | reference gene | *MdEF1α (elongation factor 1 α)* |
| EF1α_TM2 | GATGACCTGAGCGATAAAGTTGGC |  |  |  |  |
| ACT7-FW | TGAGTCACACTGTGCCAATC | 99.0 | 60°C | reference gene | *MdACT7 (actin 7)* |
| ACT7-REV | TTTCCCGTTCAGCAGTAGTG |  |  |  |  |
| EF1b-FW | GAGAGTGGGAAATCCTCTG | 100.4 | 60°C | reference gene | *MdEF1β* (elongation factor1-beta2-like) |
| EF1b-REV | ACCAACAGCAACCAATTTC |  |  |  |  |
| UBE210-FW | AATCTCGGCTACCTGGTCAG | 98.4 | 60°C | reference gene | *MdUBE210 (ubiquitin-conjugating enzyme E210-like)* |
| UBE210-REV | GCATATGGACTGTCTGGAGGA |  |  |  |  |
| TUBB-FW | TTCTCTGGGAGGAGGTACTG | 96.0 | 60°C | reference gene | *MdTUBβ (tubulin beta chain)* |
| TUBB-REV | GTCGCATTGTAAGGCTCAAC |  |  |  |  |
| MdHDR(ex1)-FW | GGGTTGGAGCTCAGAGAACG | 126.1 | 59°C | target gene | *MdHDR* |
| MdHDR(ex1)-REV | CGGCGTCATTTAGGGTGACA |  |  |  |  |
| MdHDR(ex1/2)-FW | GGTCACCAGAGTGATAGAATTAC | 96.9 | 59°C | target gene | *MdHDR* |
| MdHDR(ex1/2)-REV | AGGGTTGATAGGAGCAGCCA |  |  |  |  |
| **Primer pairs for *HDR* qPCR analysis in transgenic *Arabidopsis thaliana* lines** | | | | |  |
| HDR_all_qPCR-FW | TCGTGGGGCAGTGCATTATT | 95.9 | 61°C | target gene | *all MdHDR variants (transgenic)* |
| HDR_all_qPCR-REV | CCACCCAGAAGACCCTTGAC |  |  |  |  |
| ACT1_2_FW | GGCGATGAAGCTCAATCCAAACG | 95.1 | 61°C | reference gene | *AtACT1(Cheng et al., 2022)* |
| ACT1_2_REV | GGTCACGACCAGCAAGATCAAGACG |  |  |  |  |
| TUB2_2_FW | GAGCCTTACAACGCTACTCTGTCT | 96.2 | 64°C | reference gene | *AtTUB2 (Cheng et al., 2022)* |
| TUB2_2_REV | ACACCAGACATAGTAGCAGAAATCA |  |  |  |  |
| TIP41-FW | TGAACTGGCTGACAATGGAGTG | 100.7 | 62°C | reference gene | *AtTIP41* (Cheng et al., 2022) |
| TIP41-REV | CATGAGCTTGGCATGACTCTCAC |  |  |  |  |
| **Primer for cloning *HDR* variants** | |  |  |  |  |
| MdHDR_FL | GATCCATGGCGTCTCTGGTTCGATCAG | |  | cloning | *MdHDR* |
| MdHDR_FS | CATGGCGTCTCTGGTTCGATCAG | |  | cloning | *MdHDR* |
| MdHDR-∆N_FL | GATCCATGGCTTTCTCCACGACCTTATC | |  | cloning | *MdHDR* |
| MdHDR-∆N_FS | CATGGCTTTCTCCACGACCTTATC | |  | cloning | *MdHDR* |
| MdHDR-sp_FL | GATCCATGTTGGCTGCTCCTATCAACCC | |  | cloning | *MdHDR* |
| MdHDR-sp_FS | CATGTTGGCTGCTCCTATCAACCC | |  | cloning | *MdHDR* |
| MdHDR_RL | AATTCTTAAAGCTGAAAATTGATAACTTGTTTCGG | |  | cloning | *MdHDR* |
| MdHDR_RS | CTTAAAGCTGAAAATTGATAACTTGTTTCGG | |  | cloning | *MdHDR* |

Table is continued on next page

Table continued from previous page

| **Name** | **Oligo sequence (5'-3')** | **qPCR efficiency [%]** | **Ta** | **Purpose** | **Gene/Reference** |
| --- | --- | --- | --- | --- | --- |
| **Other primer pairs** |  |  |  |  |  |
| EF1a-FW | ATTGTGGTCATTGGYCAYGT |  |  | control RT-PCR (cDNA) | *EF1α (elongation factor 1 α) (Boudichevskaia et al., 2009)* |
| EF1a-FW | CCAATCTTGTAVACATCCTG |  |  |  |  |
| NptII_F | ACAAGATGGATTGCACGCAGG |  |  | amplification transgene | *NptII* |
| NptII_R | AACTCGTCAAGAAGGCGATAG |  |  |  |  |
| CaMV35S_F | CCCACTATCCTTCGCAAGACCC |  |  | amplification transgene | *MdHDR variants (transgenic)* |
| BA_HDRoT_REV | GCCCTGTCCCTTATTATGTT |  |  |  |  |
| 35S-P-FW_2 | GACGTAAGGGATGACGCACA |  |  | amplification transgene | *MdHDR expression cassette* |
| Actin2-T-REV | AAAACGCAAAACGAAAGCGG |  |  |  |  |
| HDR_all(2)_FW | TCGTGGGGCAGTGCATTATT |  |  | RT-PCR | *MdHDR variants (transgenic)* |
| HDR_all(2)_REV | CCACCCAGAAGACCCTTGAC |  |  |  |  |
| Md_ges_nf1 | CGAGATTGAAAAATGGCGTCTCTGG |  |  | RT-PCR | *MdHDR* |
| 1_MD_ges_f | TCTCTGGTTCGATCAGCTACTCTC |  |  | RT-PCR | *MdHDR* |
| Anker_rev_1 | AAGCAGTGGTATCAACGCAGAGTAC |  |  | RT-PCR | *MdHDR* |
| oligo(dT) anchor | AAGCAGTGGTATCAACGCAGAGTAC (T)_23_VN |  |  | cDNA synth. | *-* |

**Table S3: LC/ESI-QTOFMS identification of tested substrates and obtained products in enzyme assays with recombinant *Md*HDR.** Depending on the nature of the substrate, either negative [M-H]^-^ or positive [M-H]^+^ ion mode was used. All substrates and products (if any) were considered positively identified when the detected masses matched the calculated masses. In the combined HDR/CHS/CHI assays, both phloretin and naringenin were obtained as products, with their ratio to one another depending on the velocity of the HDR reaction. *p*-Dihydrocoumaroyl-CoA is converted to phloretin, whereas the unconsumed *p*-coumaroyl-CoA is converted to naringenin chalcone (by CHS) and further to naringenin (either by CHI or spontaneously). When hydroxycinnamic acid CoA esters (*p*-coumaroyl-CoA, caffeoyl-CoA, feruloyl-CoA, and sinapoyl-CoA) were used as substrates, the CoA was removed by alkaline cleavage (Ibdah et al., 2014) prior to LC/MS analysis. Thus, substrates and potential products are similar to assays where the corresponding free acids are used, but the latter were not accepted as substrates. Apart from hydroxycinnamic acids and their CoA esters, a few further substrates were tested that had also been used in the characterization of another *Md*DBR (Caliandro et al., 2021; Ibdah et al., 2014). However, *p*-hydroxycinnamaldehyde, *p*‑hydroxybenzalacetone, 3-methoxy-4-hydroxy-benzalacetone, benzalacetone, all tested free hydroxycinnamic acids, as well as naringenin chalcone (Yauk et al., 2024) were not accepted as substrates. Thus, *Md*HDR shows a higher specificity for hydroxycinnamic acid CoA esters than the double bond reductase of Caliandro et al. (2021), which was shown to use *p*-coumaraldehyde rather than *p*-coumaroyl-CoA as substrate.

| Compound | m/z [M-H]^-^ calculated | m/z [M-H]^-^ detected | | Mass error  (Δ ppm) | | RT (min) |
| --- | --- | --- | --- | --- | --- | --- |
| Naringenin^C, P^ (C_15_H_12_O_5_) | 271.0612 | 271.0604 | | -2.95 | | 8.902 |
| Phloretin^C, P^ (C_15_H_14_O_5_) | 273.0768 | 273.0768 | | 0.00 | | 8.804 |
| *p*-Coumaric acid^S^ (C_9_H_7_O_3_) | 163.0401 | 163.0398 | | -1.84 | | 6.711 |
| *p*-Dihydrocoumaric acid^P^ (C_9_H_9_O_3_) | 165.0557 | 165.0555 | | -1.21 | | 6.449 |
| Caffeic acid^S^ (C_9_H_8_O_4_) | 179.0350 | 179.0347 | | -1.68 | | 5.677 |
| Dihydrocaffeic acid^P^ (C_9_H_10_O_4_) | 181.0506 | 181.0492 | -7.73 | | 5.407 | |
| Ferulic acid^S^ (C_10_H_10_O_4_) | 193.0506 | 193.0504 | | -1.04 | | 7.114 |
| Dihydroferulic acid^P^ (C_10_H_12_O_4_) | 195.0663 | 195.0569 | | -2.05 | | 6.810 |
| Sinapinic acid^S^ (C_11_H_12_O_5_) | 223.0612 | 223.0610 | | -0.89 | | 7.084 |
| Dihydrosinapinic acid^P^ (C_11_H_14_O_5_) | 225.0768 | 225.0766 | | -0.90 | | 6.760 |
| Naringenin chalcone^S^ (C_15_H_12_O_5_) | 271.0612 | 271.0609 | | -1,11 | | 8.661 |
| Phloretin^P^ (C_15_H_14_O_5_) | 273.0768 | Not detected |  | |  | |
| *p*-Coumaraldehyde^S^ (C_9_H_8_O_2_) | 147.0452 | 147.0456 | | 2.72 | | 7.655 |
| *p-*Dihydrocoumaraldehyde^P^ (C_9_H_10_O_2_) | 149.0608 | Not detected |  | |  | |
| *p*-Hydroxybenzalacetone^S^ (C_10_H_10_O_2_) | 161.0608 | 161.0611 | | 1.86 | | 8.112 |
| Raspberry ketone^P^ (C_10_H_12_O_2_) | 163.0765 | Not detected |  | |  | |
| 3-Methoxy-4-hydroxy-benzalacetone^S^ (C_11_H_12_O_3_) | 193.0859 | 193.0864 | | 2.59 | | 8.467 |
| 3-Methoxy-4-hydroxy-phenylaceton^P^ (C_11_H_14_O_3_) | 195.1016 | Not detected |  | |  | |
| Compound | **m/z [M+H]^+^ calculated** | **m/z [M+H]^+^ detected** | | **Mass error  (Δ ppm)** | | **RT (min)** |
| *trans*-4-Phenyl-3-buten-2-one^S^ (C_10_H_10_O) | 147.0804 | 147.0806 | | 1.36 | | 11.184 |
| *trans*-4-Phenyl-3-butan-2-one^P^ (C_10_H_12_O) | 149.0961 | Not detected |  | |  | |

^C^ product obtained from combined HDR/CHS/CHI assay

^S^ substrate

^P^ product

**Table S4: Prediction of translation initiation sites (TISs) in *MdHDR* mRNA.** Several ATG translation initiation codons were predicted in the mRNA sequence of *MdHDR*. Their position in the mRNA, the corresponding Kozak similarity score (Gleason et al., 2022) and the reading frame of the start codon in relation to the canonical start codon (ATG at position 79) is presented.

| **Position in mRNA** | **Kozak similarity score** | **frame** |
| --- | --- | --- |
| **79** | **0.79** | **1** |
| 131 | 0.60 | 2 |
| **313** | **0.66** | **1** |
| 495 | 0.78 | 3 |
| 563 | 0.60 | 2 |
| 653 | 0.76 | 2 |
| 790 | 0.60 | 1 |
| 821 | 0.63 | 1 |
| 928 | 0.72 | 1 |
| 946 | 0.60 | 1 |
| 1026 | 0.74 | 1 |
| 1114 | 0.71 | 1 |
| 1215 | 0.76 | 3' UTR |

**Supplemental Material and Methods**

## **Chemicals**

PreScission Protease and GSTrap FF 5 mL columns were purchased from GE Healthcare (Vienna, Austria). Methanol (≥ 99.95%, for LC/MS) and acetonitrile (≥ 99.95%, for LC/MS) were purchased from Th. Geyer (CHEMSOLUTE, Renningen, Germany). Ultra-pure water (resistivity ≥ 18.2 MΩ cm) was obtained from a water purification system (Sartorius, Arium 611, Göttingen, Germany). Naringenin, naringenin chalcone, *p*-coumaroyl-CoA, feruloyl-CoA, sinapoyl-CoA and caffeoyl-CoA were purchased from Transmit (Gießen, Germany), *p-*coumaraldehyde and 3-methoxy-4-hydroxy-benzalacetone from Biosynth (Bratislava, Slovakia), *p*-hydroxybenzalacetone from Fisher Scientific (Vienna, Austria), ferulic acid from Fluka (Vienna, Austria),
*p*-dihydrocoumaric acid from Alfa-Aesar (Vienna, Austria), Sigma-Aldrich (Vienna, Austria) or Merck (Vienna, Austria), dihydroferulic acid and dihydrocaffeic acid from Alfa-Aesar (Vienna, Austria) or Sigma-Aldrich (Vienna, Austria), NADP and formic acid (≥99.0% for LC/MS) was purchased from Roth (Karlsruhe, Germany). Phloretin, malonyl-CoA, NADPH, NADH, caffeic acid, *p*-coumaric acid, glucose 6‑phosphate dehydrogenase and standard chemicals were obtained from Sigma-Aldrich (Vienna, Austria). Ultrapure water was obtained using a Direct-Q®3UV Millipore Water Purification System (Merck, Vienna, Austria). Primers were ordered from Sigma-Aldrich (Vienna, Austria) and Biomers.net (Ulm, Germany).

## **Plant material and plant growth condition**

For protein purification, plant material of *M.* × *domestica* cv. 'Golden Delicious' was collected in the experimental orchard of the University of Natural Resources and Life Sciences in Vienna (Austria). Young terminal leaves were harvested 1–2 times per week during the springs of 2015–2017, in the growing period starting from end of flowering (BBCH stage 70) to T-Stage (BBCH stage 74). The collected leaves with a length of approximately 3 cm, a light green colour, and no visible pathogenic alterations (such as fungus infections, lesions or insect and spider eggs) were shock-frozen immediately in the field with liquid nitrogen and kept at -80 °C until use. For plant transformation experiments, the *A. thaliana* ecotype Col-0 was used. For reproduction of the plant lines, plants were grown in soil (1:3 mixture of Brill Substrate Typ 3 and Vermiculit) under long day conditions (16 h light/8 h dark at 20 °C). For sterile plant culture, seeds were surface sterilized with 70% ethanol and 4% sodium hypochlorite solution, followed by rinsing with ddH_2_O. After overnight stratification at 4 °C, the sterilized seeds were plated on selective germination medium (GM: 4.9 g/L Murashige & Skoog medium, 10 g/L saccharose, pH 5.7, 7.6 g/L plant agar) and incubated in a plant growth chamber (Percival Scientific, Perry, IA, USA; CU-36L4; 16 h light at 22 °C/8 h dark at 20 °C). Plant material for gene expression analysis of the *M.* × *domestica* cultivars 'Gala', 'Pinova', 'Braeburn' and 'Topaz' was harvested from trees cultivated in the experimental field of the Julius Kühn Institute in Dresden-Pillnitz (Germany).

## **Enzyme assays**

The HDR assays according to Ibdah et al. (2014) contained in a final volume of 50 µL: 30 µL enzyme preparation, 5 µL 2 mM *p‑*coumaroyl‑CoA dissolved in water, 0.3 µL 20 mM NADP, 0.1 µL glucose 6‑phosphate dehydrogenase (0.0175 U) and 14.6 µL NADPH regenerating solution (0.5 µmol MgCl_2_, 1 µmol glucose 6‑phosphate dissolved in 0.3 M sodium citrate buffer pH 6). After incubation for 60 min at 30 °C, the reaction was stopped by addition of 20 µL 5 M NaOH. The reaction mixture was incubated for 45 min at 45 °C to saponify the CoA esters. Saponification was stopped by addition of 25 µL HCl conc. and the free acids were extracted with ethyl acetate twice (80 µL and 50 µL). The upper phases were transferred to a fresh tube and the solvent was completely evaporated inside a vacuum desiccator. Products and educts were dissolved in 30 µL 50% methanol and 4 µL were subjected to HPLC analysis on a Dionex UltiMate® 3000 RSLC System with DAD-3000RS Photodiode Array Detector (Thermo Scientific, Vienna, Austria) with an Acclaim™ RSLC 120 C18, 2.2 µm, 120Å, 2.1 x 150 mm (Dionex Bonded Silica Products: No. 071399) operated at 25 °C. The elution solvents were ddH_2_O with 0.1% formic acid (A) and acetonitrile with 0.1% formic acid (B). *p‑*Coumaric acid and *p‑*dihydrocoumaric acid were eluted with a flow rate of 0.2 mL/min according to the gradient (-10–0 min: 9% B; 0–30 min, 9–90% B; 30–40 min, 90% B), detected at 280 nm, identified by their retention times and UV-VIS spectra from 190 to 800 nm, and the concentrations were calculated from the peak areas of samples and the corresponding standards.

The combined HDR/CHS/CHI assay (Gosch et al., 2009) including a NADPH regeneration system contained in a ﬁnal volume of 120 µL: 25 µL recombinant HDR, 54 µL buffer (0.1 M Tris–HCl pH 7.0 containing 0.4% (w/v) sodium ascorbate), 5 µL *p*-coumaroyl-CoA (1.0 nmol), 5 µL NADPH (50 nmol), 5 µL glucose 6-phosphate and 1 µL glucose 6-phosphate dehydrogenase (0.175 U). After incubation for 20 min at 30 °C, 10 µL recombinant CHS (from *M.* *× domestica*; lab collection), 10 µL recombinant CHI (from *Petunia hybrida*; lab collection) and 5 µL Malonyl-Coenzyme A (1.5 nmol) were added. Including CHI was necessary to determine naringenin formation from remaining *p*-coumaroyl-CoA not converted to *p*-dihydrocoumaroyl-CoA by the HDR. The enzyme reaction was incubated for additional 30 min at 30 °C and stopped with 200 µL ethyl acetate and 10 µL 100% acetic acid. The total amount of products formed was quantiﬁed by measuring 100 µL of the organic phase on a scintillation counter (Perkin Elmer, Shelton, CT, USA). The residual organic phases were transferred to a pre-coated cellulose plate (Merck, Vienna, Austria). After developing the TLC plates in 15% acetic acid, the ratio phloretin/naringenin was determined with a TLC linear analyzer (Berthold, Bad Wildbad, Germany).

## **Protein extraction and removal of pigments**

The previously published protocol (Molitor et al., 2015) was adapted and optimized. The final procedure was as follows: Leaves were pulverized with an electric household blender in the presence of liquid nitrogen. 600 g frozen leaf powder was ground for 5 min with 300 g quartz sand and 1200 mL 125 mM sodium citrate buffer pH 6.5, 10% (v/v) Triton X-114, 0.4% (w/v) sodium ascorbate using a mortar and pestle. The mixture was centrifuged for 30 min at 30000 × g and 12 °C. The supernatant, which consisted of two liquid phases, was decanted and centrifuged again for 30 min at 30000 × g and 12 °C. All further steps were performed at 4 °C. The two phases of the supernatant were separated. The detergent-rich phase, possessing a higher density, was extracted twice with the same volume of ice cold 125 mM sodium citrate buffer pH 6.5, 0.4% (w/v) sodium ascorbate. All detergent-poor phases were pooled and ammonium sulphate was added to a final concentration of 50% saturation. After centrifugation for 20 min at 30000 × g and 4 °C, the pellet was dissolved in ice cold 125 mM sodium citrate buffer pH 6.5, 0.4% (w/v), sodium ascorbate and the solution was subjected to five cycles of polyethylene glycol (PEG)/ammonium sulphate extraction for polyphenol removal. In each cycle, PEG 4000 was dissolved to a concentration of 7% (w/v) with five minutes of stirring, followed by ammonium sulphate to a concentration of 30% saturation and centrifugation at 5400 × g and 4 °C for 20 min. The upper PEG phase was discarded, and the remaining suspension, along with the pellet, was retained and subjected to the next cycle. During the five cycles, the strong yellow color of the upper PEG phase faded into a very light yellow, indicating the separation of the bulk of polyphenols and their degradation products. The collected pellets were dissolved in ice cold 125 mM sodium citrate buffer pH 6.5, 0.4% (w/v), sodium ascorbate, and after addition of ammonium sulphate to a final 50% saturation, the proteins were precipitated over night at 4 °C.

## **LC/ESI-QTOFMS product identification**

LC/MS analyses for products of the combined HDR/CHS/CHI assay (Gosch et al., 2009) and the substrates and products from Table S3 were performed on an Agilent 1290 Infinity II System consisting of a binary pump (G7120A), a diode array detector (G7117C), a multisampler (G7167G) and a column compartment (G7116B) coupled with a high resolution mass spectrometer (Agilent Accuracy MS 6545 Q-TOF with dual AJS ESI Ion Source) (Agilent Technologies, Santa Clara, CA, USA). The column Agilent ZORBAX Eclipse Plus C18 Rapid Resolution HD 2.1 x 150 mm, 1.8 micron (Agilent, P.N. 959759-902) protected by a guard column UHPLC Guard ZORBAX® Eclipse Plus C18, 2,1 x 5 mm, 1,8 Micron (Agilent: P.N. 821725-901) was used. For data evaluation, MassHunter Workstation Qualitative Analysis 10.0 Software (Agilent Technologies, Inc., Santa Clara, USA) was used. 0.1% (v/v) formic acid in water and 0.1% (v/v) formic acid in acetonitrile were eluent A and B, respectively. The following gradient program at a flow rate of 300 µL/min was applied for products of the combined HDR/CHS/CHI assay: 0–12.67 min, linear from 5 to 71% B; 12.67–13.17 min, linear to 99% B; 13.17–19.17 min, isocratic, 99%; 19.17–19.50 min, linear to 5% B; 19.50–21.83 min, isocratic, 5% B; postrun 5.5 min, isocratic, 5% B. For the substrates and products from Table S3, the following gradient program at a flow rate of 300 µL/min. was applied: 0–11 min, linear from 2 to 53% B; 11–13.50 min, linear to 98% B; 13.50–19.50 min, isocratic, 98%; 19.50–20 min, linear to 2% B; 20–21 min, isocratic, 2% B; postrun 6 min, isocratic, 2% B. The column temperature was maintained at 35 °C. Injection volume was 5 µL. Eluting compounds were detected with the diode array detector at 290 nm (combined HDR/CHS/CHI assay) or 276 nm (compounds of Table S3) and in an *m/z* range of 100–1000. Mass spectra were recorded using an acquisition rate of 2 (compounds of Table S3) or 4 (combined HDR/CHS/CHI assay) spectra per second.

## **Protein purification by Fast Protein Liquid Chromatography (FPLC)**

All chromatographic purification steps were carried out using an Äkta Purifier (GE Healthcare, Vienna, Austria) in a climate-controlled room at 15 °C. The protein pellet obtained from the ammonium sulphate precipitation was redissolved in 200 mL of 30 mM MES buffer pH 6.5 containing 0.5% w/v sodium ascorbate and centrifuged at 28000 × g and 4 °C for 45 min. The supernatant was further diluted with 15 mM MES buffer pH 6.5 until the conductivity of the protein solution was below 8 mS/cm, and the diluted sample was applied to a SP Sepharose Fast Flow (Pharmacia, Uppsala, Sweden) column (HiScale 26/40, 70 mL bed volume) with an Äkta prime system pump. After washing, proteins were eluted by an increasing sodium chloride gradient over 15 column volumes up to 0.75 M sodium chloride at a flow rate of 5 mL/min. The elution fractions were then tested for HDR activity and due to the large number of fractions, 50 µL of three adjacent fractions were pooled and 30 µl of these solutions were used for the activity assays. All fractions that exhibited activity were pooled and used for the next chromatography step after concentrating and desalting them at 5400 × g and 4 °C in a Vivaspin 20 centrifugal concentrator MWCO 10 kDa (Sartorius, Göttingen, Germany). The resulting concentrated protein solution was subjected to three cation exchange Mono S 5/50 GL chromatography steps (MonoS1–S3): After dilution in 30 mL 30 mM MES buffer pH 6.5 and centrifugation at 5400 × g and 4 °C for 45 min, the supernatant was diluted with 15 mM MES buffer pH 6.5 and transferred to a preequilibrated Mono S 5/50 GL column (MonoS1). After washing, the proteins were eluted with 30 mM MES, 1 M NaCl, pH 6.5 in a linear gradient. Fractions with the highest activity were concentrated in a Vivaspin 20 centrifugal concentrator MWCO 10 kDa. After addition of 30 mL 40 mM HEPES, pH 7.2 and centrifugation at 5400 × g and 4 °C for 45 min, the supernatant was further diluted by 20 mM HEPES, pH 7.2, transferred to the preequilibrated Mono S 5/50 GL column, and elution was performed using 40 mM HEPES, 500 mM NaCl, pH 7.2 in a linear gradient (MonoS2). Finally, the active selected fractions were subjected to a last purification step following the above-described procedure but with 60 mM acetate, pH 5.6 buffer and elution was performed with a buffer containing 60 mM acetate, 500 mM NaCl, pH 5.6 in a linear gradient (MonoS3). The active fractions were concentrated (Vivaspin 500 10000 MWCO) and stored at 4 °C.

## **Molecular mass determination and protein identification**

SDS-PAGE was performed in a Mini-PROTEAN® Tetra Vertical Electrophoresis Cell (Bio-Rad Laboratories, Feldkirchen, Germany) according to the method of Laemmli (1970), using Color Prestained Protein Standard, Broad Range, 11–245 kDa (New England Biolabs, Frankfurt am Main, Germany) as molecular weight marker. Samples were incubated 1:6 with a 6 x concentrated Laemmli buffer at 95 °C for 10 minutes, loaded onto a SDS-polyacrylamide (PAA) gel composed of a 5% PAA stacking gel (pH 6.8, 10% SDS) and a 12% PAA running gel (pH 8.8, 10% SDS) and run in SDS buffer (0.025 M Tris, 0.192 M Glycin, 0.1% SDS, pH 8.3) at a constant voltage of 180 V for 35 min. Gels were stained with Coomassie Brilliant Blue R-250 (Sigma-Aldrich, Vienna, Austria).

**Cloning of *Md*HDR into pGEX-6P-1**

For cloning of *Md*HDR variants via sticky-end PCR (Zeng, 1998), the primers MdHDR_FL and MdHDR_RS (PCR 1) and MdHDR_FS and MdHDR_RL (PCR 2) were used. For cloning of MdHDR-∆N the primers MdHDR-∆N_FL and MdHDR_RS (PCR 1) and MdHDR-∆N_FS and MdHDR-RL (PCR 2) were used. For cloning of the shortened HDR proteoform (MdHDR-spl) starting at an alternative downstream methionine (Figure 6), the primers MdHDR-spl_FL and MdHDR_RS (PCR 1) and MdHDR-spl_FS and MdHDR-RL (PCR 2) were used. MdHDR-spl was subcloned in pTrcHis2-TOPO (Invitrogen, Waltham, MA, USA) before it was transferred into pGEX-6P-1, by using MdHDR in pGEX-6P-1 instead of cDNA as template. After transformation into *E. coli* TOP10, plasmids were isolated and the presence of the insert confirmed by sequencing (Microsynth, Vienna, Austria).

## **Establishment of transgenic *A. thaliana* lines**

After T-DNA transformation, transgenic plants were screened by plating seeds on solid GM medium supplemented with 100 mg/L kanamycin and 500 mg/L cefotaxim. Seeds were then collected from individual plants. In the T2 generation, plant lines were analyzed for segregation on selective GM medium to identify lines with single-locus T-DNA integration using statistical analysis (3:1 segregation [critical value χ2 (1;0.95)]). Plants with homozygous T-DNA integration were selected in the T3 generation on selective medium, and seeds were collected for further experiments.

## **cDNA synthesis and qPCR for gene expression analysis**

One µg of RNA was used for cDNA synthesis, performed with the oligo(dT)_18_ primer and the RevertAid First-Strand cDNA Synthesis Kit (Thermo Fisher Scientific, Schwerte, Germany). The resulting cDNA was diluted 1:10 for subsequent PCRs (RT-PCR, qPCR). To confirm successful cDNA synthesis and exclude genomic DNA contamination, the cDNA was used as a template in a standard RT-PCR amplifying the reference gene *EF1α* with the consensus primer pair EF1a-FW/REV (Boudichevskaia et al., 2009). PCR products were analyzed by gel electrophoresis using 1% TAE agarose gel.

For qPCR, 2 μL of the diluted cDNA were used for amplification on a CFX96 Touch real-time PCR detection system (Bio-Rad Laboratories, Feldkirchen, Germany). The reaction was performed in a total volume of 20 μL, consisting of 10 μL 2 × Maxima SYBR Green/Fluorescein qPCR Master Mix (Thermo Fisher Scientific, Schwerte, Germany), 1 μL of each primer (10 pmol/μL), and 6 μl of ddH_2_O. Following initial denaturation at 95 °C for 5 min, 36 to 41 amplification cycles (denaturation 95 °C for 10 s, annealing at T_a_ for 30 s, elongation at 72 °C for 30 s, including signal measurement) were performed. A melt curve analysis was conducted to validate product specificity for each sample. The PCR efficiency was determined in each run by a standard curve (10-fold dilution), which was also used for inter-run calibration. Data analysis and gene expression studies were conducted using the CFX Maestro™ Software (Bio-Rad Laboratories, Feldkirchen, Germany). The selection of stably expressed reference genes for normalization, the integrated tool of the CFX Maestro™ Software or the web-tool RefFinder (www.ciidirsinaloa.com.mx/RefFinder-master) was used. Gene expression analyses were carried out with three technical replicates for each sample.

**HDR target enrichment for PacBio long read sequencing**

RNA isolation and cDNA synthesis was performed as described in the previous section using the oligo(dT) anchor primer. For target enrichment, proof reading PCRs (Q5 High-Fidelity DNA Polymerase, New England Biolabs, Frankfurt am Main, Germany) were performed with the primers Md_ges_nf1 and Anker_rev_1 and cDNA as a template, followed by a nested PCR with the primers 1_MD_ges_f and Anker_rev_1 (Table S2). The reverse primer Anker_rev_1 binds to the anchor sequence which was introduced during cDNA synthesis by the oligo(dT) anchor. After gel electrophoresis using 1% TAE agarose gel (Figure S9a), products between 1 and 2 kb of four PCR experiments were cut out, purified, pooled and used for PacBio long read sequencing. Quality parameters of the sample were determined spectrophotometrically (NanoDrop 2000 (Thermo Scientific, Schwerte, Germany); A_260/280_: 1.80, A_260/230_: 2.41; 106 ng/µL; Figure S9b, c).

## **Processing of plant material and extract preparation for metabolite analysis**

Deep-frozen plant material was dried for two days using a freeze dryer (Gamma 1-16 LSC, Christ, Osterode am Harz, Germany; ice condenser temperature -50 °C, pressure 0.04 mbar, single-chamber method). Dried plant material was transferred into a 2 mL microcentrifuge tube and homogenized (60 s, 30 Hz) using a steel ball (∅ 5 mm) and a mixer mill (MM300, Retsch, Haan, Germany). An aliquote of the resulting homogenate (15 mg ± 1 mg) was weighed into a new 2 mL microcentrifuge tube. Afterwards, 600 µL methanol/water, 8/2 (v/v) were added. The resulting mixture was vortex-mixed (10 s), sonicated (5 min, 37 kHz, bath temperature 25–30 °C) and shaken (60 min, 2000 rpm, 22 °C). After centrifugation (13000 × g, 10 min, 22 °C) a 200 µL aliquot of the supernatant was transferred into an HPLC microvial. A further 20 µL aliquot of the supernatant was used to prepare a pooled quality control (QC) sample. All extracts were stored in a fridge at 6 °C until analysis.

## **RP-UHPLC/ESI-QTOFMS**

LC/MS analyses of *Arabidopsis* samples were performed on an Infinity 1290 series UHPLC system (Agilent Technologies, Santa Clara, CA, USA) consisting of a binary pump (G4220A), an autosampler (G4226A, 20 µL loop), an autosampler thermostat (G1330B) and a thermostatted column compartment (G1316C) which was coupled with an iFunnel Q-TOF mass spectrometer (G6550A, Agilent Technologies, Santa Clara, CA, USA) via a dual Agilent jet stream electrospray ion source. MassHunter LC/MS Data Acquisition software was used for controlling the instrument and data acquisition as well as MassHunter Qualitative and Quantitative Analysis software for data evaluation. The mass spectrometer was operated in low mass range (*m/z* 1700) and extended dynamic range (2 GHz) mode. The instrument was auto tuned and calibrated according to manufacturer´s recommendations using ESI-L tuning mix (Agilent Technologies, Santa Clara, CA, USA). Reference mass correction was used throughout all experiments. For this purpose, a solution of purine (37.5 µM) and hexakis-(2,2,3,3-tetrafluoropropoxy)phosphazine (37.5 µM) in acetonitrile/water, 95/5 (v/v) was continuously introduced through the second sprayer of the dual ion source at a flow rate of 18 µL/min using an external HPLC pump equipped with a 1:100 splitting device. Extracts (1.5 µL) were separated on a Zorbax RRHD Eclipse Plus C18 column (100 mm × 2.1 mm, 1.8 µm particle size, Agilent Technologies, Santa Clara, CA, USA) using 0.1% (v/v) formic acid in water and 0.1% (v/v) formic acid in acetonitrile as eluent A and B, respectively. The following binary gradient program at a flow rate of 400 µL/min was applied: 0–15 min, linear from 5 to 95% B; 15–18 min, isocratic, 95% B; 18–20 min, isocratic, 5% B. The column temperature was maintained at 40 °C and the autosampler temperature at 6 °C. Eluting compounds were detected in an *m/z* range of 70–1700 in negative ion mode. Centroid mass spectra were recorded using an acquisition rate of 3 spectra per second. For further instrument settings see Tais et al. (2021).

Collision-induced dissociation (CID) mass spectra were acquired in targeted-MS^2^ mode using scheduled precursor ion lists and the following parameters: acquisition rate MS, 3 spectra per second; acquisition rate MS/MS, 3 spectra per second, isolation width, narrow (1.3 *m/z*); collision energy, 10–30 V; collision gas, nitrogen.

## **References**

Boudichevskaia, A., Flachowsky, H. and Dunemann, F. (2009) Identification and molecular analysis of candidate genes homologous to HcrVf genes for scab resistance in apple. *Plant breeding* **128**, 84-91.

Caliandro, R., Polsinelli, I., Demitri, N., Musiani, F., Martens, S. and Benini, S. (2021) The structural and functional characterization of Malus domestica double bond reductase MdDBR provides insights towards the identification of its substrates. *International Journal of Biological Macromolecules* **171**, 89-99.

Cheng, S.-S., Ku, Y.-S., Cheung, M.-Y. and Lam, H.-M. (2022) Identification of stably expressed reference genes for expression studies in Arabidopsis thaliana using mass spectrometry-based label-free quantification. *Frontiers in plant science* **13**, 1001920.

Gleason, A.C., Ghadge, G., Chen, J., Sonobe, Y. and Roos, R.P. (2022) Machine learning predicts translation initiation sites in neurologic diseases with nucleotide repeat expansions. *PLoS One* **17**, e0256411.

Gosch, C., Halbwirth, H., Kuhn, J., Miosic, S. and Stich, K. (2009) Biosynthesis of phloridzin in apple (Malus domestica Borkh.). *Plant Science* **176**, 223-231.

Ibdah, M., Berim, A., Martens, S., Valderrama, A.L.H., Palmieri, L., Lewinsohn, E. and Gang, D.R. (2014) Identification and cloning of an NADPH-dependent hydroxycinnamoyl-CoA double bond reductase involved in dihydrochalcone formation in Malus× domestica Borkh. *Phytochemistry* **107**, 24-31.

Laemmli, U. (1970) Denaturing (SDS) discontinuous gel electrophoresis. *Nature* **277**, 680-685.

Molitor, C., Mauracher, S.G., Pargan, S., Mayer, R.L., Halbwirth, H. and Rompel, A. (2015) Latent and active aurone synthase from petals of C. grandiflora: a polyphenol oxidase with unique characteristics. *Planta* **242**, 519-537.

Robinson, P. (2017) Integrative genomics viewer (IGV): Visualizing alignments and variants. In: *Computational exome and genome analysis* pp. 233-245. Chapman and Hall/CRC.

Tais, L., Schulz, H. and Böttcher, C. (2021) Comprehensive profiling of semi‐polar phytochemicals in whole wheat grains (Triticum aestivum) using liquid chromatography coupled with electrospray ionization quadrupole time‐of‐flight mass spectrometry. *Metabolomics* **17**, 1-18.

Yang, S., Zong, W., Shi, L., Li, R., Ma, Z., Ma, S., Si, J., Wu, Z., Zhai, J., Ma, Y., Fan, Z., Chen, S., Huang, H., Zhang, D., Bao, Y., Li, R. and Xie, J. (2024) PPGR: a comprehensive perennial plant genomes and regulation database. *Nucleic acids research* **52**, D1588-d1596.

Yauk, Y.K., Dare, A.P., Cooney, J.M., Wang, Y., Hamiaux, C., McGhie, T.K., Wang, M.Y., Li, P. and Atkinson, R.G. (2024) Naringenin chalcone carbon double-bond reductases mediate dihydrochalcone biosynthesis in apple leaves. *Plant Physiol* **196**, 2768-2783.

Zeng, G. (1998) Sticky-end PCR: new method for subcloning. *Biotechniques* **25**, 206-208.
